# Comparative Analysis of Generative AI Language Models in Orthodontics: Evidence‐Based Insights Into Perplexity, iASK, and ChatGPT 4o Mini

**DOI:** 10.1155/tswj/5479774

**Published:** 2026-03-03

**Authors:** Simarpreet Bhamra, Ramya Vijeta Jathanna

**Affiliations:** ^1^ Department of Orthodontics and Dentofacial Orthopedics, Manipal College of Dental Sciences, Manipal Academy of Higher Education, Manipal, India, manipal.edu

## Abstract

**Objective:**

This study is aimed at evaluating and comparing the scientific reliability of three large language models (LLMs), Perplexity, iASK, and ChatGPT 4o mini, based on their responses to orthodontic‐related queries.

**Materials and Methods:**

The three LLMs were prompted with 10 clinical orthodontic questions, and their responses were assessed independently by two evaluators using a structured scoring system (0–10). Statistical analyses, including Pearson and Spearman correlations, Cronbach′s alpha, and Wilcoxon signed‐rank test, were performed to determine interevaluator reliability and model performance differences.

**Results:**

Perplexity achieved the highest mean score (7.2), followed by iASK (5.4) and ChatGPT 4o mini (5.2). High consistency between evaluators was observed (Cronbach^′^s alpha = 0.947). A significant difference was noted between Perplexity and both ChatGPT 4o mini and iASK (*p* = 0.002). Pearson and Spearman correlations indicated strong agreement between evaluators (*r* = 0.982, *ρ* = 1.000).

**Conclusion:**

Perplexity demonstrated superior performance in orthodontic‐related queries compared to ChatGPT 4o mini and iASK. The findings highlight the importance of evaluating AI models for clinical applicability and reliability.

## 1. Introduction

Artificial Intelligence (AI) is a branch of computer science that studies how well a machine can mimic human cognitive processes. Because AI may be used to tackle a wide range of jobs, the field has shown a lot of promises during the past 10 years. [1] In the field of orthodontics, AI‐based technologies and applications are advancing rapidly. This is clear from our field′s growing focus on AI‐based research [2].

Inspired by the biological neural system of the human brain, artificial neural networks (ANNs) are a subdomain of machine learning. [3] One notable application of ANNs is the analysis of complex relationships among large amounts of data. [4] Three layers are usually present in an ANN: an input layer, an output layer, and at least one hidden layer. [5] Multiple hidden layer ANNs, or “deep learning,” are widely used and have shown remarkable performance in projects involving computer vision, including segmentation and classification. [6] As deep learning is very feasible, computational performance is increasing, and sophisticated model training techniques are available, deep learning is becoming more and more popular. [7]

When it comes to orthodontic issues, AI can be used as an adjunctive tool. AI can be used in orthodontics to plan treatments and anticipate their outcomes. As an illustration, it can simulate how pre‐ and post‐treatment facial photos will look different. The use of AI algorithms has substantially improved communication between patients and dentists by enabling unambiguous visualization of the effects of orthodontic therapy, skeletal patterns, and anatomic landmarks in lateral cephalograms. [8] AI has the potential to aid in the systematic evaluation of clinically relevant scientific evidence through data synthesis, risk factor identification, and pattern recognition. When thoughtfully combined with the dentist′s clinical experience and the patient′s treatment requirements and preferences, this could help busy clinicians overcome the obstacles involved in implementing the evidence‐based dentistry (EBD) strategy for oral healthcare. [9] These AI models are thus occupying important voids in healthcare decision‐making, education, and communication as the need for quick, reliable, and easily accessible information increases.

A sophisticated AI language model named Chat‐based Generative Pre‐trained Transformer (ChatGPT) was created by Open Artificial Intelligence (San Francisco, California, United States), [10] who′s first edition launched in November 2022, following which it immediately became the application with the fastest growth rate in history, with 100 million users active as of February 2023 [11]. Specifically, ChatGPT, a large language model (LLM), is based on natural language processing (NLP), a branch of AI that aims to make it possible for computers to understand natural language inputs. [12] ChatGPT, on one hand, poses a challenge to modern LLMs by enabling students to produce essays or answers rapidly and with little research effort. [13] However, on the other hand, fake information was reported to be generated via ChatGPT, along with erroneous or biased responses on specific subjects. The chatbot′s restrictions resulting from its training data, which prevent it from knowing about recent occurrences, are another major cause for concern. Educators might take advantage of this restriction by mandating that students include current events in their writing tasks. [14]

The same year also saw the advent of Perplexity, which is a cognitive search engine that makes use of many language models. The platform′s free version mostly uses GPT‐3.5, while its subscription edition, Perplexity Pro, gives users access to GPT‐4 in addition to additional sophisticated models including Claude 3.5 and Mistral Large. Similarly, iAsk is headquartered in Chicago, Illinois, and was established in 2022. It makes use of a large‐scale transformer language‐based model, which is comparable to ChatGPT′s technology, but it has been specially trained on credible and reputable sources to deliver accurate responses. While a few recent articles have examined the validity of ChatGPT in comparison to different LLM models, such as Microsoft Bing and Google Bard, none have examined the evidence‐based effectiveness of AI models, such as Perplexity and iASK, in comparison to ChatGPT 4o mini. Thus, the objective of this study was to comparatively assess the answers generated by these LLM models, in response to clinically relevant orthodontic questions.

## 2. Materials and Methods

The abovementioned three distinct LLMs, namely, ChatGPT 4o mini, Perplexity, and iASK were asked 10 indicative questions pertaining to typical clinical concerns in orthodontics. [15] All queries were performed using the **free web-based versions** of each platform to simulate real‐world clinical use by general practitioners without subscription access. Because commercial AI platforms dynamically route user prompts across available backend models, this study evaluated platform‐level performance rather than fixed underlying model weights. The specific platform configurations at the time of data collection were as follows:

• **Perplexity AI:** Free web version with automatic model selection enabled by the platform (default Sonar/Llama‐based models with potential dynamic routing to other models such as GPT‐4o mini depending on system allocation).
• **iASK AI:** Free publicly accessible web version utilizing its proprietary transformer‐based language model available at the time of query.
• **ChatGPT:** Free OpenAI interface using the **GPT-4o mini** model.

All queries were performed without user login personalization where possible, to minimize adaptive learning effects and maintain uniform testing conditions across platforms. The platforms were considered distinct based on their interface architecture, response generation systems, and user interaction environments rather than assumed independence of underlying foundational models.

Ten clinically relevant orthodontic questions were developed through author consensus and verified against established scientific literature and professional guidelines to serve as reference standards. Verification of references cited within LLM responses was performed independently by the evaluators using peer‐reviewed orthodontic literature and academic databases, and unverifiable citations negatively influenced scientific accuracy scoring. Each platform received identical single‐prompt queries without follow‐up clarification to simulate rapid chairside information retrieval. All the information obtained served as the “gold standard” against which the responses from the LLMs were assessed (Table S1).

The three independent LLMs were asked these open‐ended questions using appropriate jargons by any one of the authors, who could pose each LLM the question only once, without reiterating extra explanation, or follow‐up questions if the LLM was unable to respond. This way it reflected current reality by replicating instances in which oral healthcare practitioners demand prompt help with single enquiries. Furthermore, restricting interactions to single questions made it possible to assess the LLMs′ capacity to respond succinctly and pertinently to intricate questions without the need for repeated prompts, allowing the procedure to be one‐time and quick.

Based on an assessment criterion, [16] each AI model′s responses were evaluated independently by the two authors and given a score between 0 (minimum) and 10 (maximum). To combat prejudice, three LLM models were given numbers as part of a double blinding technique. These models were additionally assessed for scientific accuracy, comprehensiveness, clarity, and relevance in addition to interevaluator reliability. The following null hypothesis was assessed: there is no significant difference between ChatGPT 4o mini, Perplexity, and iASK based on answers obtained via each of them.

### 2.1. Statistical Analysis

Statistical analysis was performed using IBM SPSS Statistics (Version 29.0; IBM Corp., Armonk, New York, United States). The data were summarized by calculating indices of central tendency and indices of variability for average scores. Interevaluator reliability was tested using Cronbach′s alpha. Statistical analysis was done to determine which model provided answers with the highest levels of comprehensiveness, scientific accuracy, clarity, and relevance. Statistical comparisons were conducted on the scores using Wilcoxon′s test. Additionally, Pearson and Spearman correlation analyses were conducted to assess agreement between evaluators and to evaluate the relationship between comprehensiveness and scientific accuracy of responses. In every hypothesis and testing method, the significance level was established at *a* = 0.05 (*p* < 0.05).

## 3. Results

Ten orthodontic clinical queries were evaluated across three generative AI platforms: Perplexity, iASK, and ChatGPT 4o mini (Table 1). Responses were assessed using four predefined domains: comprehensiveness, scientific accuracy, clarity, and clinical relevance.

**TABLE 1:** Orthodontic queries used for LLM evaluation.

Q no	Questions
Q1	Does orthodontic treatment affect the airway function?
Q2	Is early orthodontic treatment in two phases for children with prominent upper teeth more beneficial compared to treatment that is provided in one phase in adolescence?
Q3	Do light vibrational forces reduce the duration of orthodontic treatment?
Q4	How long should one wait to go for surgical exposure after an interceptive intervention for palatally displaced permanent canine?
Q5	What is the best orthodontic treatment for posterior crossbites?
Q6	How do molar tubes compare to molar bands in terms of failure and decalcification?
Q7	Do painkillers, taken before or after orthodontic treatment, help relieve pain? If so, which painkillers work best?
Q8	Are removable better than fixed retainers?
Q9	Does extracting wisdom teeth (third molars) affect crowding later in life?
Q10	Which wires work best for initial tooth alignment?

Table 2 indicates that all three models showed very high consistency between the evaluators (Cronbach^′^s alpha > 0.9), so an average score for every LLM was computed. Table 3 depicts the average evaluation scores across queries.

**TABLE 2:** Cronbach′s *a* for the scores given by the two evaluators to the answers provided by the three LLMs.

LLM [evaluators 1–2]	Cronbach′s alpha
ChatGPT	0.962
Perplexity	0.938
Ask AI	0.999

**TABLE 3:** Average evaluation scores across queries.

Q no	LLM	C	ScA	CL	R
Q1	ChatGPT 4o mini	6.5	4.0	4.5	6.0
	Perplexity	9.0	6.5	8.0	8.0
	iASK	8.0	6.5	8.0	8.0
Q2	ChatGPT 4o mini	7.0	5.5	8.0	8.0
	Perplexity	6.5	8.0	8.0	8.0
	iASK	8.0	6.5	8.0	7.0
Q3	ChatGPT 4o mini	8.0	7.0	6.0	5.0
	Perplexity	7.0	7.5	7.0	7.0
	iASK	4.0	3.5	3.0	5.0
Q4	ChatGPT 4o mini	6.0	4.0	6.0	7.0
	Perplexity	5.0	6.0	7.0	7.0
	iASK	6.0	4.0	3.5	7.0
Q5	ChatGPT 4o mini	3.0	3.0	3.0	3.5
	Perplexity	6.5	8.0	8.0	8.0
	iASK	2.0	3.5	4.0	5.0
Q6	ChatGPT 4o mini	4.0	1.5	5.0	5.0
	Perplexity	5.5	7.0	4.0	5.0
	iASK	3.0	5.0	4.0	5.0
Q7	ChatGPT 4o mini	5.0	4.0	4.0	5.0
	Perplexity	7.0	7.0	6.0	7.0
	iASK	6.0	5.0	5.0	7.0
Q8	ChatGPT 4o mini	8.0	3.5	8.0	7.0
	Perplexity	8.0	7.0	8.0	8.0
	iASK	8.0	5.0	8.0	8.0
Q9	ChatGPT 4o mini	2.0	3.5	3.0	3.0
	Perplexity	7.0	8.0	6.0	7.0
	iASK	6.0	5.5	5.0	4.0
Q10	ChatGPT 4o mini	5.0	4.0	7.0	7.0
	Perplexity	8.0	9.0	8.0	8.0
	iASK	3.0	4.0	4.0	3.0

Abbreviations: C, comprehensiveness; CL, clarity; R, relevance; ScA, scientific accuracy.

Descriptive statistics of average score is depicted in Table 4.

**TABLE 4:** Descriptive statistics for the scores given by the two evaluators to the answers provided by the three LLMs.

	ChatGPT		Perplexity		Ask AI	
Evaluator	1	2	1	2	1	2
Min	3	3	5	6	4	4
Median	5.5	6	7.5	7.5	5	5
Max	7	7	8	8	8	8
Mean	5.20	5.30	7.20	7.20	5.40	5.40
SEM	0.467	0.473	0.327	0.291	0.476	0.476
SD	1.476	1.494	1.033	0.919	1.506	1.506
CO‐v	2.178	1.067	2.267	2.044	0.844	2.269

Abbreviations: CoV, coefficient of variance; Max, maximum; Min, minimum; SD, standard deviation; SEM, standard error of mean.

The LLM answers scoring the highest were those of Perplexity (average score = 7.2), followed by iASK (average score = 5.4), and finally ChatGPT 4o mini (average score = 5.25).

**FIGURE 1:**
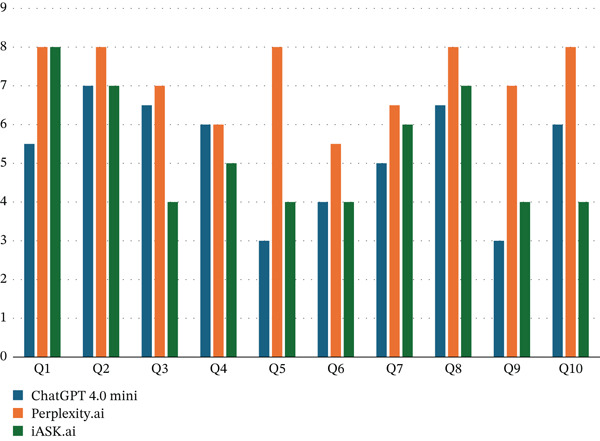
The average scores for the answers to each question provided by the three LLMs.

The average scores for the answers to each question provided by the three LLMs are represented in Figure 1. A statistically significant difference was noted between the average scores for ChatGPT 4o mini and perplexity (*p* value = 0.002), and Perplexity and iAsk (*p* value = 0.002) (Table 5).

**TABLE 5:** Wilcoxon′s *p* value for the scores given by the two evaluators to the answers provided by the three LLMs.

LLM [evaluators 1–2] average scores	Wilcoxon test *p*value
ChatGPT vs. Perplexity	0.002∗
Perplexity.ai vs. Ask AI	0.002 ∗
ChatGPT vs. Ask AI	0.414

∗Statistically significant.

A Spearman correlation analysis was conducted to see if more comprehensive responses tend to be more scientifically accurate. It was observed that for ChatGPT 4o mini and iASK, more comprehensive answers are generally more scientifically accurate, but this relationship is not observed in Perplexity (Table 6).

**TABLE 6:** A Spearman correlation analysis between comprehensiveness and scientific accuracy evaluators to the answers provided by the three LLMs.

Model	Correlation (*ρ*)	*p*value
ChatGPT	0.64	0.002∗
Perplexity.ai	0.17	0.461
iASK.ai	0.72	0.0003∗

∗Statistically significant.

Perplexity performed the best overall, scoring highest in scientific accuracy (7.4) and relevance (7.3). The platform achieved higher mean scores in treatment‐planning and appliance‐based queries (Q5, Q8, Q10), suggesting stronger alignment with evidence‐based orthodontic principles. ChatGPT 4o mini and iASK had similar performance, but ChatGPT 4o mini was slightly better in relevance. Scientific accuracy showed the biggest difference between models, where Perplexity significantly outperformed the others.

ChatGPT 4o mini produced responses with good structural organization and clarity; however, variability in scientific accuracy was observed. Lower scores were noted in clinically nuanced questions requiring precise biomechanical interpretation (Q5, Q6, Q9), indicating limitations under single‐prompt conditions without iterative clarification.

iASK demonstrated moderate performance overall, with acceptable comprehensiveness for general orthodontic concepts but comparatively lower scientific accuracy in complex biomechanical and diagnostic scenarios (Q3, Q10). Responses were typically concise but occasionally lacked detailed evidence‐based justification.

Across all models, performance varied depending on the complexity of the query. Conceptual and general orthodontic knowledge questions yielded higher scores, whereas clinical decision‐making and case‐based scenarios demonstrated greater interplatform variability.

## 4. Discussion

The definition of EBD, a subset of Evidence‐Based Medicine (EBM), is as follows: “an approach to oral health care that requires the judicious integration of systematic assessments of clinically relevant scientific evidence, relating to the patient′s oral and medical condition and history, with the dentist′s clinical expertise and the patient′s treatment needs and preferences” [17]. Professional and scientific oral healthcare organizations work to integrate EBD into dental clinical practice by developing and disseminating clinical practice guidelines. However, implementation of EBD is hindered by persistent issues such as rapid advancements in science and technology, out‐of‐date guidelines, insufficient evidence as well as workflow in practice. [18] This problem was overcome with the introduction of newest generation of GenAI chatbots that seems to have the potential to be a perfect tool for the effective application and improvement of EBD. These chatbots can theoretically instantly generate evidence‐based answers to scientific queries, functioning as the dentist′s chairside scientific advisor.

When comparing findings with previous studies evaluating generative AI in healthcare, it is important to consider that commercial platforms dynamically route prompts across multiple backend models. In the present study, Perplexity responses were generated using the free web interface with automatic model selection (typically Sonar/Llama‐based models with potential dynamic routing), whereas iASK utilized its proprietary publicly accessible model. ChatGPT 4o mini responses were generated using the GPT‐4o mini model. Therefore, the observed differences reflect real‐world platform performance rather than fixed underlying model architectures. Perplexity frequently provided reference citations within its responses; however, given the known risk of hallucinated or fabricated references in LLMs, all cited sources were independently verified by the evaluators against peer‐reviewed orthodontic literature and established academic databases. References that were incomplete, unverifiable, or inconsistent with current scientific evidence resulted in lower scientific accuracy scores. These findings highlight the importance of clinician‐led verification before integrating AI‐generated references into clinical decision‐making.

This study is therefore aimed at examining modern capabilities of ChatGPT 4o mini, Perplexity, and iASK, with an emphasis on the precision and effectiveness of the responses produced in response to queries related to Orthodontics. Through the responses, it was seen that Perplexity scored the highest (avg score = 7.2), followed by iASK (avg score = 5.4), lastly ChatGPT 4o mini. This result is contrary to those obtained in a study done by Antonietta et al., [19] which indicated that ChatGPT 3.5 overpowered Perplexity in terms of education related to gastroenterology.

While ChatGPT′s accuracy varies depending on input and conversational context, Perplexity′s accuracy is reinforced by its ability to cite sources for the information it provides, which solidifies its use case in research and academia. Additionally, ChatGPT is designed to provide plausible responses based on patterns in its training data, which frequently results in inaccurate responses. According to a previous study, ChatGPT 4o mini showed the highest accuracy for knowledge‐based questions, while Copilot had the lowest [20]. However, in our study when compared to ChatGPT 4.0 mini, Perplexity showed the highest scientific accuracy (7.40). Perplexity also showed highest comprehensiveness (6.95) contrary to study conducted by comparing Perplexity to Google Bard in which the latter showed the highest comprehensiveness. [21]

### 4.1. Limitations

Several limitations should be considered when interpreting the findings of this study. First, the performance of generative AI platforms is inherently influenced by prompt formulation and query structure. Variations in wording, level of detail, and contextual framing may significantly affect the completeness and scientific accuracy of generated responses. Although standardized single‐prompt queries were used in the present study to maintain methodological consistency and enable direct comparison between platforms, real‐world clinical users may formulate questions differently, potentially resulting in variations in response quality [22].

Second, while efforts were made to design clinically relevant orthodontic questions based on expert consensus and established references, the selected queries may not represent the full spectrum of orthodontic clinical scenarios. Standardization of query design is essential for improving reproducibility and ensuring that clinicians working in dental and orthodontic settings obtain consistent and comparable AI‐generated responses [23].

Finally, the dynamic nature of commercial AI platforms, including periodic updates and backend model routing, may influence response characteristics over time, limiting the generalizability of findings to future platform versions [24].

Future studies should investigate structured clinical prompt templates to improve consistency, reliability, and safe integration of AI‐assisted decision support in orthodontic practice.

## 5. Conclusion

An emerging area of significance is the clinical relevance of LLMs in the field of orthodontics. Our research offers evidence‐based insights into their capabilities, applications, and potential benefits to the field, contributing valuable information to the ongoing discussions around AI and its role in healthcare. The study includes a detailed comparison of the models′ accuracy, efficiency, and reliability, of which Perplexity demonstrated superior performance in orthodontic‐related queries compared to ChatGPT 4o mini and iASK. These AI‐driven tools can support decision‐making, provide evidence‐based recommendations, and facilitate enhanced patient communication. By leveraging LLMs, orthodontists can access real‐time clinical guidance, improve diagnostic accuracy, and streamline administrative processes, ultimately enhancing patient outcomes.

While our study offers valuable insights, further research is needed to assess the long‐term impact of emerging trends in orthodontics. Ultimately, this work reinforces the necessity of continuous improvement and adaptation in orthodontics, fostering a generation of well‐equipped professionals ready to meet the evolving needs of oral healthcare.

## Funding

No funding was received for this manuscript.

## Conflicts of Interest

The authors declare no conflicts of interest.

## Supporting information


**Supporting Information** Additional supporting information can be found online in the Supporting Information section. The 10 clinically relevant orthodontic questions used in this study are listed in Table S1, along with the reference standards used for validation. Expert consensus was used to formulate these questions, which were then confirmed by reference to accepted scientific literature and professional guidelines. To improve methodological transparency, reproducibility, and clarity regarding the evaluation process of the three generative AI platforms, the supplemental material offers thorough documentation of the query framework.

## Data Availability

The data that support the findings of this study are available on request from the corresponding author. The data are not publicly available due to privacy or ethical restrictions.
