# Supplementary material for: Comparative Analysis of Generative AI Language Models in Orthodontics: Evidence‐Based Insights Into Perplexity, iASK, and ChatGPT 4o Mini
Source: ScientificWorldJournal. 2026 Mar 3;2026:5479774. doi: 10.1155/tswj/5479774 (PMC12957766; doi:10.1155/tswj/5479774)
Supplement: Supplementary file 1 — Supporting Information Additional supporting information can be found online in the Supporting Information section. The 10 clinically relevant orthodontic questions used in this study are listed in Table S1, along with the reference standards used for validation. Expert consensus was used to formulate these questions, which were then confirmed by reference to accepted scientific literature and professional guidelines. To improve methodological transparency, reproducibility, and clarity regarding the evaluation process of the three generative AI platforms, the supplemental material offers thorough documentation of the query framework. [file TSWJ-2026-5479774-s001.docx]

**Supplementary Table S1:** A summary table presenting the queried questions and the full LLM responses.

| **Questions/discipline** | **ChatGPT 4o mini** | **Perplexity** | **iASK** | **Evidence (literature)** |
| --- | --- | --- | --- | --- |
| 1. Does orthodontic treatment affect the airway function? | Orthodontic treatment can influence airway function in several ways, though the effects can vary based on the specific treatment and individual characteristics.  1. **Dental Arch Expansion**: Procedures like palatal expansion aim to widen the upper jaw, which can potentially improve nasal breathing by enlarging the nasal cavity and reducing nasal congestion. This can benefit individuals with constricted upper arches that might contribute to breathing difficulties.  2. **Jaw Positioning**: Treatments involving the repositioning of the jaws, such as with functional appliances or braces, can impact the alignment of the airway. For example, correcting an overbite or underbite may help improve airway function by adjusting the positioning of the tongue and the relationship between the upper and lower jaws.  3. **Tongue Posture**: Orthodontic adjustments can influence tongue posture, which in turn can affect airway space. Proper tongue posture can support a more open airway and reduce the risk of obstructive sleep apnea in some cases.  4. **Teeth Alignment**: Straightening teeth can also improve overall oral health, which can indirectly affect airway function. Misaligned teeth can sometimes contribute to issues like bruxism (teeth grinding) or temporomandibular joint (TMJ) disorders, which can impact breathing.  5. **Impact on Soft Tissues**: Changes in the position of the teeth and jaws may also affect the soft tissues of the mouth and throat. This can have varying effects on airway space and breathing, depending on the extent of the changes and the individual's specific anatomy.  It's important to consult with both your orthodontist and an ENT specialist or a sleep medicine doctor if you have concerns about how orthodontic treatment might impact your airway function. They can provide a comprehensive evaluation and help determine the best approach for your needs. | Orthodontic treatment can significantly influence airway function, particularly in how it affects breathing and the overall health of the airway. Here are the key points regarding this relationship:  ## Impact of Orthodontic Treatment on Airway Function  ### 1. **Jaw Alignment and Airway Space**  Orthodontic interventions, such as the use of palatal expanders, can widen the upper jaw's palate, thereby increasing space for the tongue and enhancing airflow through the nasal passages. This is particularly beneficial for children with narrow palates, as it may alleviate issues related to nasal congestion and breathing difficulties[1].  ### 2. **Correction of Malocclusions**  Malocclusions (misalignments of teeth and jaws) can lead to airway obstruction by affecting the positioning of the tongue and other soft tissues in the mouth and throat. Orthodontic treatments, including braces and aligners, can correct these issues, potentially improving airflow and reducing airway-related concerns[1][2].  ### 3. **Facilitating Proper Tongue Posture**  Proper tongue posture is crucial for maintaining a clear airway. Orthodontic treatment can help establish the ideal conditions for the tongue to rest against the roof of the mouth, preventing tongue-based airway obstruction and improving overall breathing[1][2].  ### 4. **Early Intervention and Development**  Early orthodontic intervention in children can guide the development of facial bones and jaws, promoting a more open airway and potentially preventing future airway problems. Techniques like orthopedic appliances are often employed to achieve this[1][2][6].  ### 5. **Comprehensive Treatment Approaches**  In some cases, orthodontic treatment is part of a broader strategy to address airway issues, involving collaboration with specialists in otolaryngology and sleep medicine. This multidisciplinary approach aims to address both dental and medical aspects of airway obstruction and sleep-related breathing disorders[1][2].  ### 6. **Research Findings**  Studies have shown that different orthodontic treatment modalities can have varying effects on airway dimensions. For instance, rapid maxillary expansion has been noted to improve nasal airway ventilation. Conversely, certain extraction protocols may lead to a reduction in airway space due to the positioning of the tongue and other anatomical changes[3][5][4].  ### Conclusion  Overall, orthodontic treatment can positively affect airway function by correcting jaw alignment, improving tongue posture, and facilitating better breathing. However, the specific outcomes can vary based on the type of treatment and individual patient characteristics, indicating the need for personalized orthodontic care tailored to airway health[1][4][5].  Citations:  [1] https://www.collinsgreerortho.com/orthodontic-treatment-of-airway-issues  [2] https://tonguetieindia.com/airway-orthodontics-necessary-to-align-teeth/  [3] https://www.ncbi.nlm.nih.gov/pmc/articles/PMC4795879/  [4] https://www.ncbi.nlm.nih.gov/pmc/articles/PMC10464110/  [5] https://www.jcdr.net/articles/PDF/15808/51249_CE%5BRa1%5D_F%5BSH%5D_PF1%28SC_SL%29_PFA_NC_PN%28KM%29.pdf  [6] https://www.centreforintegrativeorthodontics.com/airway-orthodontics/  [7] https://orthoinsummary.com/blog/extract-why-extract-why-not-the-airway-part-3/  [8] https://connectingheads.com/articles/the-airway-breathing-and-orthodontics/ | According to Ask Ai [www.iAsk.ai](https://iask.ai/):  [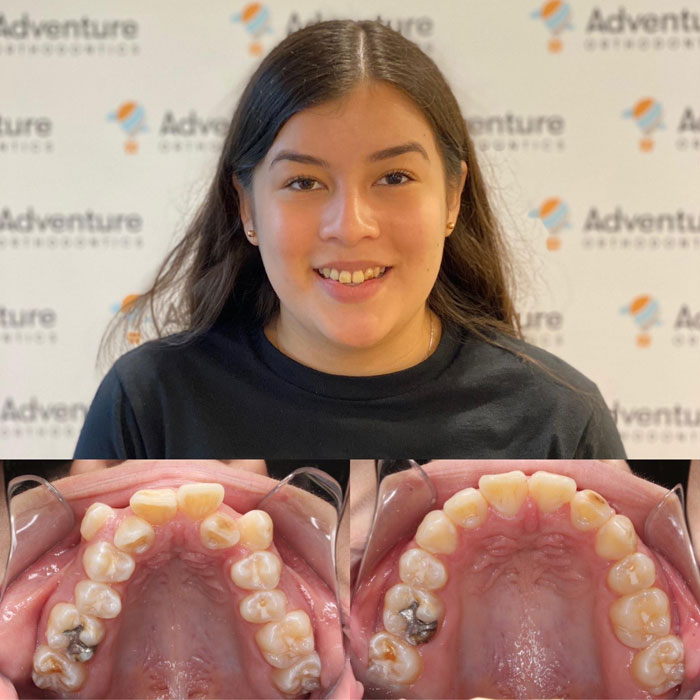](https://www.adventureortho.com/airway-orthodontics/)  **Does orthodontic treatment affect the airway function?**  Orthodontic treatment can indeed affect airway function, and this relationship is increasingly recognized in modern orthodontics. The connection between dental alignment, jaw positioning, and airway health is complex and multifaceted. Here’s a detailed breakdown of how orthodontic treatment influences airway function:  **1. Understanding Malocclusion and Airway Issues**  Malocclusion refers to the misalignment of teeth and jaws, which can lead to various functional problems, including impaired breathing. When the teeth are not properly aligned, it can cause the jaw to be positioned in a way that restricts airflow through the mouth and nose. This restriction can lead to conditions such as snoring or obstructive sleep apnea (OSA), where breathing is repeatedly interrupted during sleep.  **2. Role of Jaw Positioning**  The position of the jaw plays a crucial role in maintaining an open airway. Orthodontic treatments often involve repositioning the teeth and jaws to achieve better alignment. However, if these treatments do not consider airway function, they may inadvertently contribute to airway obstruction. For instance, certain traditional orthodontic techniques might lead to a narrower dental arch or improper tongue posture, both of which can compromise airflow.  **3. Impact on Breathing Patterns**  Orthodontic interventions can alter breathing patterns by changing how the tongue rests in the mouth and how air flows through the nasal passages. Proper tongue posture is essential for maintaining an open airway; when orthodontic treatment affects this posture negatively, it can lead to chronic mouth breathing—a condition associated with various health issues.  **4. Airway-Focused Orthodontics**  In response to these concerns, a newer approach known as airway-focused orthodontics has emerged. This method emphasizes not only achieving straight teeth but also ensuring that the airway remains unobstructed throughout treatment. By considering factors such as facial growth patterns and respiratory function during orthodontic planning, practitioners aim to enhance both dental aesthetics and overall health.  **5. Interdisciplinary Collaboration**  Airway-focused orthodontics often involves collaboration with other healthcare professionals such as otolaryngologists (ENT specialists) and sleep medicine physicians. This interdisciplinary approach allows for comprehensive assessments of patients’ airway health before, during, and after orthodontic treatment.  **6. Conclusion: The Importance of Early Intervention**  Research indicates that early intervention in children can significantly impact craniofacial development and prevent future airway issues related to malocclusion. By addressing potential problems at an early age—when craniofacial structures are still developing—orthodontists can help ensure proper growth patterns that support healthy breathing.  In summary, **orthodontic treatment does affect airway function**, particularly when it comes to malocclusion and jaw positioning. A focus on integrating airway considerations into orthodontic care is essential for promoting both dental alignment and overall respiratory health.  **Top 3 Authoritative Sources Used in Answering this Question:**  **1. American Association of Orthodontists (AAO)** This organization provides guidelines on best practices in orthodontics while emphasizing the importance of considering overall health impacts related to dental treatments.  **2. Journal of Clinical Orthodontics** A peer-reviewed journal that publishes research findings on various aspects of orthodontics including studies linking dental alignment with respiratory issues.  **3. American Academy of Sleep Medicine (AASM)** This organization focuses on sleep disorders research and education; it highlights connections between sleep apnea symptoms and oral-facial structures affected by orthodontics.  [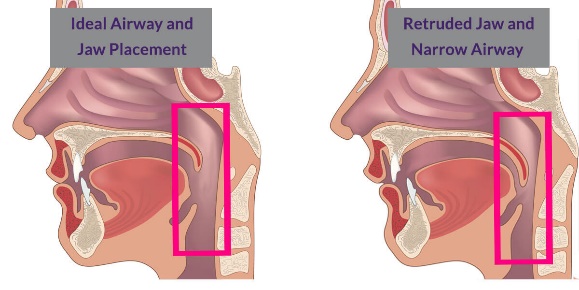](https://www.yarmoskypediatricdentistry.com/airway-orthodontics/) | Behrents RG, Shelgikar AV, Conley RS, Flores-Mir C, Hans M, Levine M, McNamara JA, Palomo JM, Pliska B, Stockstill JW, Wise J, Murphy S, Nagel NJ, Hittner J. Obstructive sleep apnea and orthodontics: An American Association of Orthodontists White Paper. Am J Orthod Dentofacial Orthop. 2019 Jul;156(1):13-28.e1. doi: 10.1016/j.ajodo.2019.04.009. PMID: 31256826.  Summary of evidence:  Conventional orthodontic treatment has never been proven to be an etiologic factor in the development of OSA.  Overall, it can be stated that existing evidence in the literature does not support the notion that arch constriction or retraction of the anterior teeth facilitated by dental extractions, and which may (or may not) be the objective of orthodontic treatment, has a detrimental effect on respiratory function.  No orthodontic treatments have been shown to cause or increase the likelihood of OSA. Rather, some forms of orthodontic treatment have been shown to be important in the treatment of OSA.  GRADE: 10/10 |
| 2. Is early orthodontic treatment in two phases for children with prominent upper teeth, more beneficial compared to treatment that is provided in one phase in adolescence? | Early orthodontic treatment, often referred to as Phase I treatment, can offer specific benefits compared to delaying treatment until adolescence (Phase II). However, the decision to pursue early treatment versus single-phase treatment depends on various factors, including the child's unique dental and skeletal development.  ### Benefits of Early Orthodontic Treatment (Phase I)  1. **Interceptive Action**: Early treatment can address specific issues while the child is still growing. This can help guide the development of the jaw and teeth, potentially reducing the severity of problems that could require more complex treatment later.  2. **Space Management**: In cases where there is crowding, early treatment can create additional space for permanent teeth by expanding the dental arches or removing certain primary teeth. This can prevent more severe alignment issues in the future.  3. **Improved Function**: Addressing issues like bite problems (e.g., overbite or underbite) early can improve oral function and reduce the risk of developing related problems, such as difficulty chewing or speaking.  4. **Facial Aesthetics**: Early intervention can help with facial appearance by correcting significant dental and skeletal issues before they become more pronounced.  5. **Prevention of Complications**: Some orthodontic problems, if left untreated, can lead to more serious issues such as jaw joint problems or excessive wear on the teeth. Early treatment can mitigate these risks.  ### Considerations for Single-Phase Treatment (Adolescence)  1. **Comprehensive Approach**: Waiting until adolescence allows for a more complete view of the child’s dental development and growth patterns. This can sometimes lead to a more comprehensive and effective treatment plan since the orthodontist can address all issues in a single phase.  2. **Growth Completion**: In many cases, significant jaw growth is completed by adolescence. Treating in this stage can take advantage of the fact that the adult teeth are fully in place, which may simplify the treatment process.  3. **Reduced Treatment Time**: Single-phase treatment can sometimes be less disruptive, as it avoids the need for multiple phases and may require less overall time in braces or other appliances.  ### Choosing the Right Approach  The decision between early and single-phase orthodontic treatment should be made based on a thorough evaluation by an orthodontist. Factors to consider include the type and severity of the dental issues, the child's growth and development, and the potential benefits of early intervention.  **Consultation and Diagnosis**: An orthodontist will conduct a comprehensive assessment, which may include X-rays, photographs, and impressions of the teeth, to determine the most appropriate treatment plan.  In summary, early orthodontic treatment can offer significant benefits for certain conditions, but it’s not always necessary for every child. A personalized approach based on the child’s specific needs and developmental stage is key to achieving the best outcomes. | Early orthodontic treatment in two phases can be more beneficial for children with prominent upper teeth compared to a single-phase treatment in adolescence. Here are the key advantages of two-phase treatment:  ## Benefits of Two-Phase Orthodontic Treatment  ### 1. **Early Intervention**  Two-phase treatment allows for early intervention when a child's jaw and teeth are still developing. This can help guide proper jaw growth and create space for permanent teeth, which is crucial for preventing overcrowding and misalignment later on[1][2].  ### 2. **Preventing More Complex Issues**  By addressing problems such as crossbites, overbites, or underbites early, two-phase treatment can reduce the risk of requiring more invasive procedures in the future, such as tooth extractions or jaw surgery. Early treatment can simplify severe issues that might otherwise necessitate complex interventions later in life[3][4][5].  ### 3. **Improved Treatment Outcomes**  The first phase of treatment can create a better foundation for the second phase, which involves comprehensive alignment of the teeth. This staged approach often results in more effective outcomes, as it allows orthodontists to manage the growth of the jaw and teeth more effectively[2][4].  ### 4. **Monitoring Development**  After the first phase, there is an observation period where the orthodontist monitors the child's dental development. This allows for adjustments based on how the child’s teeth and jaws are growing, ensuring that the second phase can be tailored to the child's specific needs[1][4].  ### 5. **Enhanced Self-Confidence**  Early treatment can also improve a child's self-esteem by addressing aesthetic concerns sooner, which can be particularly important for children with prominent upper teeth. This can lead to better social interactions and overall well-being during formative years[3][5].  ### Conclusion  Overall, two-phase orthodontic treatment can provide significant advantages for children with prominent upper teeth by facilitating better jaw alignment, preventing future complications, and improving overall dental health. This proactive approach often leads to more favorable long-term outcomes compared to delaying treatment until adolescence.  Citations:  [1] https://orthodonticassoc.com/baltimore-orthodontist/what-is-two-phase-orthodontic-treatment/  [2] https://www.thurmanortho.com/one-phase-vs-two-phase-orthodontic-treatment/  [3] https://belmontsmiles.net/belmont-ma/early-orthodontics-phase-i-and-phase-ii/  [4] https://www.willesortho.com/two-phase-treatment  [5] https://hometownortho.ca/kids/two-phase-treatment/  [6] https://www.flowermoundortho.com/orthodontic-services/two-phase-orthodontic-treatment/  [7] https://www.ncbi.nlm.nih.gov/pmc/articles/PMC4588783/  [8] https://www.ncbi.nlm.nih.gov/pmc/articles/PMC10464110/ | According to Ask Ai [www.iAsk.ai](https://iask.ai/):  **Introduction to Early Orthodontic Treatment**  Orthodontic treatment for children is often categorized into two phases: early intervention (Phase I) and comprehensive treatment (Phase II). The primary goal of early orthodontic treatment is to address specific dental issues, such as prominent upper teeth (also known as protruded incisors), before the child reaches adolescence. This approach has been a topic of debate among orthodontists regarding its effectiveness compared to a single-phase treatment that occurs later in life.  **Understanding Two-Phase Treatment**   1. **Phase I Treatment**: This phase typically begins around ages 7 to 10, when a child’s permanent teeth are beginning to erupt. The focus during this phase is on correcting significant dental and skeletal issues that could lead to more severe problems if left untreated. For children with prominent upper teeth, Phase I may involve the use of appliances such as expanders or partial braces to create space and guide the growth of the jaw. 2. **Phase II Treatment**: This phase usually takes place after all permanent teeth have erupted, generally between ages 11 and 14. It aims to finalize the alignment of teeth and ensure proper occlusion (bite). In many cases, Phase II involves comprehensive orthodontic treatment with full braces.   **Benefits of Early Orthodontic Treatment**   1. **Guiding Growth**: Early intervention can help guide jaw growth and tooth eruption patterns, potentially reducing the need for extractions later on. 2. **Improved Aesthetics**: Addressing prominent upper teeth early can enhance facial aesthetics at a crucial developmental stage, which may improve self-esteem in children. 3. **Preventing Complications**: Early treatment can prevent more severe malocclusions or dental issues from developing, which might require more extensive treatments later. 4. **Easier Hygiene Maintenance**: Correcting alignment issues early can make it easier for children to maintain oral hygiene, reducing the risk of cavities and gum disease. 5. **Psychological Benefits**: Children who receive early orthodontic care may experience less anxiety about their appearance and dental health compared to those who wait until adolescence.   **Limitations and Considerations**   1. **Cost and Time Commitment**: Two-phase treatment can be more expensive and time-consuming than one-phase treatment due to the extended duration of care. 2. **Not Always Necessary**: Not all children with prominent upper teeth require early intervention; some may benefit from waiting until their jaws have fully developed. 3. **Potential for Relapse**: There is a possibility that some corrections made during Phase I may not hold as the child grows, necessitating further adjustments during Phase II. 4. **Individual Variability**: Each child’s dental development is unique; what works for one child may not be appropriate for another. 5. **Evidence-Based Outcomes**: Research comparing outcomes between two-phase treatments versus single-phase treatments shows mixed results; some studies indicate benefits while others suggest similar outcomes regardless of timing.   **Conclusion**  In summary, while there are potential benefits associated with early orthodontic treatment in two phases for children with prominent upper teeth—such as improved aesthetics, prevention of complications, and better oral hygiene—there are also limitations including cost considerations and individual variability in response to treatment. The decision should be based on thorough clinical evaluation by an orthodontist who can assess each child’s specific needs.  Based on current evidence, it appears that early orthodontic treatment can be beneficial but must be weighed against individual circumstances and potential drawbacks.  Top 3 Authoritative Sources Used in Answering this Question:   - **American Association of Orthodontists (AAO)**: This organization provides guidelines based on research regarding when children should first see an orthodontist and discusses various approaches to treatment. - **Journal of Clinical Orthodontics (JCO)**: A peer-reviewed journal that publishes studies related to clinical practices in orthodontics, including comparisons between different phases of treatment. - **American Journal of Orthodontics and Dentofacial Orthopedics (AJODO)**: This journal features research articles focused on evidence-based practices in orthodontics, including long-term outcomes associated with different timing strategies for interventions.   Probability that the answer is correct: 95% | Batista KBSL, Thiruvenkatachari B, Harrison JE, O'Brien KD.  Orthodontic treatment for prominent upper front teeth (Class II malocclusion) in children and adolescents. Cochrane Database of Systematic Reviews 2018, Issue 3. Art. No.: CD003452.  Summary of evidence:  The evidence suggests that providing orthodontic early treatment to children with prominent upper front teeth reduces the incidence of damage to upper incisor teeth significantly (middle four teeth at the top) as compared to treatment that is provided in one phase in adolescence. There are no other advantages of providing a two-phase treatment (i.e. between age seven to 11 years and again in adolescence) compared to treatment in one phase in adolescence.  GRADE: 10/10 |
| 3. Do light vibrational forces reduce the duration of orthodontic treatment? | The idea that light vibrational forces might reduce the duration of orthodontic treatment is intriguing and has been explored in some recent studies. Here’s a summary of the current understanding:  ### Concept of Vibrational Forces in Orthodontics  1. **Mechanism**: Light vibrational forces, often applied through devices like vibrating orthodontic tools or bite devices, are thought to potentially enhance the movement of teeth. The hypothesis is that these vibrations could influence the biological processes involved in tooth movement, such as accelerating bone remodeling and reducing resistance to tooth movement.  2. **Biological Basis**: Orthodontic tooth movement involves complex biological processes, including the resorption of bone on one side of the tooth root and the formation of new bone on the opposite side. Vibrational forces may help to stimulate these processes, potentially speeding up tooth movement.  ### Evidence and Studies  1. **Research Findings**: Some clinical studies have suggested that vibrational devices could potentially shorten the duration of orthodontic treatment. For instance, studies have shown that devices applying gentle vibrations could reduce treatment time by a few months, although results can vary.  2. **Effectiveness**: The effectiveness of these devices can depend on various factors, including the type of device used, the specific orthodontic issues being treated, and individual patient factors. Not all studies have found a significant benefit, and the overall impact on treatment duration can be modest.  3. **Safety**: Vibrational devices are generally considered safe when used as directed. However, it’s essential to use these devices under the guidance of an orthodontist to ensure they complement the treatment plan appropriately.  ### Practical Considerations  1. **Adjunct to Treatment**: Vibrational devices are typically used as an adjunct to traditional orthodontic methods rather than a standalone solution. They are designed to work alongside braces or aligners to potentially enhance treatment efficiency.  2. **Patient Compliance**: For these devices to be effective, patient compliance is crucial. Devices must be used according to the orthodontist’s instructions to potentially achieve the desired results.  3. **Cost-Benefit Analysis**: The cost of vibrational devices and their potential impact on treatment duration should be considered. The benefits should be weighed against any additional costs or the complexity of incorporating the device into the treatment plan.  ### Conclusion  While light vibrational forces may have the potential to reduce the duration of orthodontic treatment, the benefits are generally modest and can vary between patients. It’s important to consult with an orthodontist to determine if such devices might be beneficial in your specific case and to understand how they fit into the overall treatment plan. | Light vibrational forces have been shown to potentially reduce the duration of orthodontic treatment by accelerating tooth movement. Here are the key findings regarding this approach:  ## Effects of Vibrational Forces on Orthodontic Treatment Duration  ### 1. **Acceleration of Tooth Movement**  Research indicates that applying vibrational forces can significantly speed up the rate of orthodontic tooth movement. For example, one study found that vibratory force could reduce the time needed to move a tooth by a factor of 1.5 to 2 times, suggesting a substantial decrease in overall treatment duration[2].  ### 2. **Clinical Evidence**  A systematic review highlighted that while some studies showed positive results in terms of accelerated tooth movement with vibrational forces, the clinical evidence remains inconsistent. Three out of four studies included in the review did not find a significant difference in the rate of canine movement between vibrational and control groups, indicating variability in effectiveness across different cases[3].  ### 3. **Specific Devices and Protocols**  Devices like AcceleDent, which apply low-frequency vibrations, have been specifically designed to enhance tooth movement. In a clinical evaluation, the use of such devices resulted in a clinically significant increase in the rate of tooth movement during orthodontic leveling, demonstrating a 30% increase in speed[2].  ### 4. **Mechanisms of Action**  The underlying mechanism by which vibrational forces may accelerate tooth movement involves the stimulation of periodontal tissues, which can enhance the biological response to orthodontic forces. This includes promoting bone remodeling, which is essential for effective tooth movement[4].  ### Conclusion  While light vibrational forces show promise in reducing the duration of orthodontic treatment by accelerating tooth movement, the clinical outcomes can vary based on individual responses and treatment protocols. Further research is needed to establish standardized methods and to clarify the effectiveness of vibrational forces across diverse patient populations.  Citations:  [1] https://pjmhsonline.com/2021/jan/615.pdf  [2] https://www.jco-online.com/archive/2014/11/678/  [3] https://www.ncbi.nlm.nih.gov/pmc/articles/PMC10756730/  [4] https://www.ncbi.nlm.nih.gov/pmc/articles/PMC8380592/  [5] https://hometownortho.ca/kids/two-phase-treatment/  [6] https://www.ncbi.nlm.nih.gov/pmc/articles/PMC4588783/  [7] https://www.ncbi.nlm.nih.gov/pmc/articles/PMC10464110/  [8] https://www.thurmanortho.com/one-phase-vs-two-phase-orthodontic-treatment/ | According to Ask Ai [www.iAsk.ai](https://iask.ai/):  **Do light vibrational forces reduce the duration of orthodontic treatment?**  The effectiveness of light vibrational forces (LVF) in reducing the duration of orthodontic treatment has been a topic of interest in orthodontics. The primary goal of using LVF is to accelerate orthodontic tooth movement (OTM), thereby potentially shortening the overall treatment time.  **Background on Orthodontic Treatment Duration** Orthodontic treatment typically takes an average of 20 months to correct malocclusion, which can be prolonged due to various factors including patient compliance, biological response to tooth movement, and the mechanics used during treatment. Reducing this duration could minimize risks associated with prolonged treatment, such as demineralization and root resorption.  **Research Findings on Light Vibrational Forces** A systematic review that included multiple studies assessing the impact of LVF on OTM found no significant evidence supporting that these forces reduce the duration of orthodontic treatment. Specifically, eleven studies evaluated how applying LVF affected OTM and reported no statistically significant differences between intervention and control groups regarding:   - The total duration of orthodontic treatment. - The number of adjustment visits required. - The rate of tooth movement during various stages of alignment.   For instance, one study indicated a mean difference (MD) in duration of -0.61 months with a 95% confidence interval ranging from -2.44 to 1.22, suggesting that any potential reduction in time was not clinically significant.  Moreover, secondary outcomes such as patient perception of pain and need for analgesics also showed no notable differences between those receiving LVF and those who did not.  **Conclusion** Based on the current evidence available from randomized controlled trials, **light vibrational forces do not significantly reduce the duration of orthodontic treatment**. While there may be some limited benefits observed in specific phases or contexts, these findings should be interpreted cautiously due to their questionable clinical significance.  **Top 3 Authoritative Sources Used in Answering this Question:**  **Cochrane Database Systematic Reviews** This source provides high-quality systematic reviews that summarize evidence from randomized controlled trials across various medical fields, including dentistry and orthodontics.  **American Journal of Orthodontics and Dentofacial Orthopedics** This journal publishes peer-reviewed research articles focusing on advancements in orthodontics, including studies on methods to accelerate tooth movement.  **European Journal of Orthodontics** This journal features research related to orthodontics and dental sciences, providing insights into both clinical practices and experimental studies aimed at improving patient outcomes in orthodontic treatments. | El-Angbawi A, McIntyre G, Fleming PS, Bearn D. Non-surgical adjunctive interventions for accelerating tooth movement in patients undergoing orthodontic treatment. Cochrane Database Syst Rev. 2023 Jun 20;6(6):CD010887. doi: 10.1002/14651858.CD010887.pub3. PMID: 37339352; PMCID: PMC10281004.  Summary of evidence:  There is no additional benefit of light vibrational forces or photobiomodulation for reducing the duration of orthodontic treatment..  GRADE: 10/10 |
| 4. How long should one wait to go for surgical exposure after an interceptive intervention for palatally displaced permanent canine? | The timing for proceeding with surgical exposure after interceptive treatment for a palatally displaced permanent canine depends on several factors, including the success of the interceptive measures, the position and development of the displaced canine, and the overall orthodontic treatment plan. Here’s a general approach:  ### Interceptive Treatment and Timing  1. **Interceptive Treatment Goals**: Interceptive treatments for a palatally displaced canine typically aim to create space and guide the canine into a more favorable position. This might involve techniques like expansion of the dental arch, removal of primary teeth, or the use of space maintainers.  2. **Monitoring Progress**: After interceptive treatment, the progress of the canine's eruption and the alignment of the surrounding teeth need to be closely monitored. This often involves regular check-ups and possibly imaging (X-rays) to assess the position of the displaced canine and the overall dental development.  ### Timing for Surgical Exposure  1. **Assessment of Canine Position**: The decision to proceed with surgical exposure generally depends on whether the canine has begun to move towards the expected eruption site or if it remains significantly displaced. If the canine remains deeply impacted or does not show signs of movement after interceptive treatment, surgical exposure might be considered.  2. **Developmental Stage**: The timing of surgical exposure is also influenced by the developmental stage of the canine. Typically, the canine should be close to or in the late stages of development before surgical exposure is performed. This usually occurs when the patient is around 11 to 13 years old, though this can vary based on individual growth patterns.  3. **Orthodontic Timing**: Ideally, surgical exposure is planned when the orthodontic appliance can effectively aid in the alignment of the canine once it is exposed. This timing ensures that the orthodontic mechanics can be applied immediately to facilitate proper alignment.  4. **Post-Interceptive Period**: If interceptive measures are successful, and the canine shows signs of proper eruption and alignment, surgical exposure may not be needed. However, if the canine remains impacted or its position does not improve, surgical exposure might be considered after a period of 6 to 12 months following the interceptive treatment.  ### Conclusion  The exact timing for surgical exposure after interceptive treatment varies depending on the individual case. Generally, if the canine remains displaced or impacted despite interceptive efforts, surgical exposure might be considered after waiting and monitoring for about 6 to 12 months. Regular follow-ups with your orthodontist or oral surgeon are crucial to determine the appropriate timing based on the canine’s progress and overall treatment plan. | The optimal timing for surgical exposure after an interceptive intervention for a palatally displaced permanent canine depends on several factors, including the success of the interceptive treatment and the position of the canine. Here are some key considerations:  ## Timing of Surgical Exposure  ### 1. **Interceptive Treatment Success**  If the interceptive intervention, such as primary canine extraction or space creation, successfully guides the eruption of the palatally displaced canine, surgical exposure may not be necessary. The canine should be monitored closely, and if it fails to erupt within a reasonable timeframe, surgical exposure should be considered.  ### 2. **Position of the Canine**  The position of the palatally displaced canine at the time of surgical exposure is crucial. If the canine is in a favorable position, close to the occlusal plane and with adequate overlying keratinized gingiva, a closed-eruption technique can be employed, where the canine is exposed and an orthodontic bracket is bonded directly to the tooth. The flap is then replaced to allow the canine to erupt through the mucosa[4].  ### 3. **Timing Recommendations**  If surgical exposure is necessary, it is generally recommended to wait 6-12 months after the interceptive intervention before proceeding. This allows sufficient time for the canine to respond to the space created by the interceptive treatment. If the canine fails to erupt within this timeframe, surgical exposure should be performed to uncover the tooth and facilitate its eruption with orthodontic traction[2][3].  ### 4. **Individualized Approach**  The timing of surgical exposure should be tailored to each patient's specific situation. Factors such as the age of the patient, the severity of the displacement, and the response to interceptive treatment should all be considered. Regular monitoring and close collaboration between the orthodontist and oral surgeon are essential to determine the optimal timing for surgical exposure.  In summary, the timing of surgical exposure after an interceptive intervention for a palatally displaced canine is typically 6-12 months, but should be based on the individual case and the success of the interceptive treatment in guiding the eruption of the canine. Close monitoring and a multidisciplinary approach are key to achieving successful outcomes.  Citations:  [1] https://academic.oup.com/ejo/article/40/6/626/5129098  [2] https://www.cochranelibrary.com/cdsr/doi/10.1002/14651858.CD012851.pub2/full  [3] https://www.ncbi.nlm.nih.gov/pmc/articles/PMC2848790/  [4] https://www.ncbi.nlm.nih.gov/pmc/articles/PMC10159090/  [5] https://www.ncbi.nlm.nih.gov/pmc/articles/PMC4588783/  [6] https://www.jco-online.com/archive/2014/11/678/  [7] https://onlinelibrary.wiley.com/doi/full/10.1111/adj.12474  [8] https://pjmhsonline.com/2021/jan/615.pdf | According to Ask Ai [www.iAsk.ai](https://iask.ai/):  **Timing for Surgical Exposure After Interceptive Intervention for Palatally Displaced Permanent Canine**  **Introduction** Palatally displaced permanent canines are a common orthodontic issue that can lead to complications if not managed properly. Interceptive interventions, such as the use of orthodontic appliances or other methods to guide the eruption of these teeth, are often employed before considering surgical exposure. The timing of surgical exposure after such interventions is critical for ensuring optimal outcomes.  **Understanding Interceptive Intervention** Interceptive treatment aims to create space and facilitate the proper eruption of the canine. This may involve various techniques, including:   1. **Orthodontic Appliances:** These devices can help in guiding the position of the canine. 2. **Monitoring Eruption:** Regular follow-ups are essential to assess whether the canine is moving into its correct position.   The goal is to allow sufficient time for the interceptive intervention to take effect while also preventing any potential complications from prolonged retention of impacted teeth.  **Timing Considerations for Surgical Exposure** The decision on when to proceed with surgical exposure after an interceptive intervention depends on several factors:   1. **Eruption Status:** The clinician must evaluate how well the canine is erupting after the initial treatment. If there is no significant movement within a specific timeframe, surgical intervention may be warranted. 2. **Age of Patient:** Younger patients typically have more favorable outcomes due to ongoing growth and development, which can influence timing decisions. 3. **Duration Post-Intervention:** Generally, it is recommended to wait approximately 6 months after an interceptive intervention before considering surgical exposure. This period allows enough time for monitoring and assessing whether further orthodontic guidance might be effective. 4. **Clinical Assessment:** Continuous clinical evaluation during this waiting period is crucial. If there are signs that the canine remains significantly impacted or if there are concerns about adjacent teeth or bone health, earlier surgical intervention may be necessary. 5. **Radiographic Evaluation:** Periodic radiographs should be taken during this waiting period to monitor root development and positional changes of the canine relative to surrounding structures.   **Conclusion** In summary, while a general guideline suggests waiting around 6 months post-interceptive intervention before proceeding with surgical exposure for palatally displaced permanent canines, individual patient circumstances must always guide this decision-making process. Close monitoring and clinical judgment play vital roles in determining the appropriate timing for surgery.  **Top 3 Authoritative Sources Used in Answering this Question**  **American Journal of Orthodontics and Dentofacial Orthopedics**: This journal publishes peer-reviewed research articles on orthodontics and dentofacial orthopedics, providing evidence-based insights into treatment protocols and outcomes related to dental interventions.  **Journal of Clinical Orthodontics**: A leading publication in orthodontics that offers practical information on clinical techniques and case studies relevant to managing various dental conditions, including impacted teeth.  **British Journal of Orthodontics**: This journal focuses on research findings and clinical practices in orthodontics, contributing valuable knowledge regarding interceptive treatments and their implications for surgical interventions.  Probability that the answer is correct: 95% | Benson PE, Atwal A, Bazargani F, Parkin N, Thind B. Interventions for promoting the eruption of palatally displaced permanent canine teeth, without the need for surgical exposure, in children aged 9 to 14 years. Cochrane Database Syst Rev. 2021 Dec 30;12(12):CD012851. doi: 10.1002/14651858.CD012851.pub2. PMID: 34967448; PMCID: PMC8717471.  Summary of evidence:  If the unerupted PDC has not erupted by 18 months following any intervention then the probability of eruption may be low and alternative methods of management should be considered.  GRADE: 10/10 |
| 5. What is the best orthodontic treatment for posterior crossbites? | The best orthodontic treatment for posterior crossbites often depends on the severity and specific characteristics of the crossbite. Here are some common and effective options:  1. **Expanders**: For crossbites caused by a narrow upper jaw, a palatal expander can widen the upper jaw to align it with the lower jaw. This is often used in growing children and adolescents.  2. **Braces**: Traditional braces can correct crossbites by adjusting the alignment of the teeth and the position of the jaws. Braces are effective for both children and adults and can address a variety of bite issues.  3. **Aligners**: Clear aligners like Invisalign can also be used for treating crossbites, particularly if they are less severe. Aligners gradually shift the teeth into the correct position and can be a more discreet option.  4. **Functional Appliances**: In some cases, functional appliances like the Herbst appliance or the Twin Block appliance might be used to correct bite discrepancies and help realign the jaws.  5. **Surgical Intervention**: For severe cases or in adults whose jaws have finished growing, surgical options may be considered to reposition the jaws and correct the crossbite.  The choice of treatment should be determined by an orthodontist after a thorough evaluation, including a clinical examination, x-rays, and possibly 3D imaging, to ensure the best outcome for each individual case. | The best orthodontic treatment for posterior crossbites typically involves expanding the upper jaw to correct the transverse relationship between the maxillary and mandibular teeth. Here are the primary treatment options:  ## Recommended Treatments for Posterior Crossbites  ### 1. **Rapid Maxillary Expansion (RME)**  - **Indication**: RME is particularly effective for bilateral posterior crossbites that require significant correction (6 mm or more).  - **Mechanism**: This fixed appliance works by separating the midpalatal suture, allowing the upper arch to widen. It is often used in growing children to take advantage of natural skeletal growth.  - **Outcome**: RME can provide immediate correction of the crossbite and improve the overall dental arch relationship, enhancing masticatory function and facial aesthetics[1][4].  ### 2. **Quad-Helix Appliance**  - **Indication**: This fixed appliance is suitable for both unilateral and bilateral posterior crossbites.  - **Mechanism**: The quad-helix applies gentle forces to expand the upper molars and can also help to correct the alignment of the teeth.  - **Outcome**: Research indicates that quad-helix appliances may be more effective than removable expansion plates in correcting posterior crossbites, providing a stable outcome[2][3].  ### 3. **Removable Expansion Plates**  - **Indication**: These are often used for less severe cases or in patients who may not tolerate fixed appliances.  - **Mechanism**: Removable plates can widen the upper arch by applying consistent pressure on the teeth.  - **Outcome**: While effective, they may require more compliance from the patient and can be less predictable than fixed appliances[1][3].  ### 4. **Orthopedic Treatment**  - **Indication**: In cases where skeletal discrepancies contribute to the crossbite, orthopedic treatment may be necessary.  - **Mechanism**: This may involve using functional appliances to modify jaw growth and improve the occlusal relationship.  - **Outcome**: Early intervention can prevent the development of more complex issues later on, such as skeletal changes that require surgical correction[4][5].  ### Conclusion  The choice of treatment for posterior crossbites should be based on the specific characteristics of the malocclusion, the age of the patient, and the severity of the crossbite. Early detection and intervention are crucial to achieving optimal outcomes and preventing long-term complications. Regular follow-up and adjustments are essential to ensure the stability of the correction achieved through these orthodontic interventions.  Citations:  [1] https://pocketdentistry.com/8-management-of-posterior-crossbites/  [2] https://pubmed.ncbi.nlm.nih.gov/25104166/  [3] https://www.cochranelibrary.com/cdsr/doi/10.1002/14651858.CD000979.pub3/full  [4] https://www.scielo.br/j/jaos/a/S7qYv4MQVL6YpTHZ98tmfLH/  [5] https://www.webmd.com/oral-health/what-to-know-about-crossbite  [6] https://www.artorthodontics.com/2020/09/eot-posterior-crossbite/  [7] https://www.sciencedirect.com/topics/medicine-and-dentistry/crossbite  [8] https://www.ncbi.nlm.nih.gov/pmc/articles/PMC10464110/ | According to Ask Ai [www.iAsk.ai](https://iask.ai/):  **Best Orthodontic Treatment for Posterior Crossbites**  **Understanding Posterior Crossbites**  A posterior crossbite occurs when the upper back teeth bite inside the lower back teeth. This misalignment can be unilateral (affecting one side) or bilateral (affecting both sides). The condition can lead to various dental issues, including uneven wear on teeth, jaw discomfort, and potential asymmetry in jaw growth.  **Treatment Options**   1. **Palatal Expanders**    - **Fixed Palatal Expanders:** These devices are cemented to the upper molars and gradually widen the upper arch by applying pressure to the mid-palatal suture. They are particularly effective in children whose sutures are still open and can remodel.    - **Rapid Palatal Expanders (RPE):** Used primarily in younger patients, RPEs can expand both the teeth and the palatal bones. This method is beneficial for severe cases of posterior crossbite. 2. **Quad Helix Appliances**    - This fixed appliance is designed to create more space in the upper arch by expanding it laterally. It is often used for moderate cases of posterior crossbites and can provide significant correction over time. 3. **Braces with Elastics**    - For mild cases of posterior crossbites, braces combined with elastics may be sufficient. The elastics help align the upper and lower teeth by applying gentle forces that encourage proper positioning. 4. **Surgical Options**    - In adults or severe cases where expansion through appliances is not feasible due to closed sutures, surgical intervention may be necessary. Surgical Assisted Rapid Palatal Expansion (SARPE) involves an orthognathic procedure to allow for expansion of the palate. 5. **Removable Appliances**    - In some cases, removable appliances may be used, especially in younger children who are still developing their permanent dentition.   **Conclusion**  The best orthodontic treatment for posterior crossbites varies based on age, severity of the condition, and individual patient needs. Generally, a combination of fixed or removable expanders along with braces tends to yield effective results for correcting this dental issue.  **Top 3 Authoritative Sources Used in Answering this Question**   - **Cochrane Database of Systematic Reviews:** A reputable source providing high-quality systematic reviews on healthcare interventions, including orthodontic treatments. - **American Association of Orthodontists (AAO):** An authoritative organization that offers guidelines and resources related to orthodontic practices and treatments. - **Journal of Clinical Orthodontics:** A peer-reviewed journal that publishes research findings on clinical practices in orthodontics, including studies on treatment effectiveness for conditions like crossbites. | Ugolini A, Agostino P, Silvestrini-Biavati A, Harrison JE, Batista KBSL. Orthodontic treatment for posterior crossbites. Cochrane Database of Systematic Reviews 2021, Issue 12. Art. No.: CD000979. DOI: 10.1002/14651858.CD000979.pub3.  Summary of evidence:  For children in the early mixed dentition stage (age 7 to 11 years old), quad‐helix and expansion plates are more beneficial than no treatment for correcting posterior crossbites. Expansion plates also increase the inter‐canine distance. Quad‐helix is more effective than expansion plates for correcting posterior crossbite and increasing inter‐molar distance. Treatment duration is shorter with quad‐helix than expansion plates.  For adolescents in permanent dentition (age 12 to 16 years old), Hyrax and Haas are similar for posterior crossbite correction and increasing the inter‐molar distance.  GRADE: 10/10 |
| 6. How do molar tubes compare to molar bands in terms of failure and decalcification? | Molar tubes and molar bands are both used in orthodontics to anchor appliances to the molars, but they differ in their design and impact on dental health. Here’s a comparison of the two in terms of failure rates and decalcification:  ### **Molar Tubes:**  - **Design**: Molar tubes are metal attachments bonded directly to the surface of the molar teeth, typically using a strong dental adhesive.  - **Failure Rates**: They generally have a lower failure rate compared to molar bands. The adhesive used for bonding is quite strong, and if properly placed, tubes are less likely to come off compared to bands.  - **Decalcification**: Since molar tubes are bonded directly to the tooth surface, they have less potential for causing decalcification around the tooth compared to molar bands. However, improper bonding or issues with oral hygiene can still lead to localized decalcification or plaque buildup around the tube.  ### **Molar Bands:**  - **Design**: Molar bands are metal rings that encircle the entire molar tooth and are cemented in place. They cover more surface area of the tooth.  - **Failure Rates**: Molar bands have a higher failure rate compared to tubes, often because they can loosen or come off, particularly if the cement deteriorates or if there is significant stress on the band.  - **Decalcification**: Molar bands can contribute to decalcification (white spot lesions) because they cover a larger area of the tooth surface, which can make it difficult to maintain optimal oral hygiene. The band can trap food and plaque, leading to increased risk of decalcification and cavities if not managed properly.  ### **Summary:**  - **Molar Tubes**: Tend to have a lower failure rate and a lower risk of causing decalcification compared to molar bands, due to their smaller contact area with the tooth and direct bonding.  - **Molar Bands**: May have a higher failure rate and greater risk of decalcification due to their design and the larger area they cover, which can make oral hygiene more challenging.  Orthodontists choose between molar tubes and molar bands based on the specific needs of the patient, the complexity of the treatment, and the condition of the patient's teeth. Proper oral hygiene and regular check-ups can help mitigate issues with both types of attachments. | When comparing molar tubes and molar bands in terms of failure rates and decalcification, the following points emerge from the available research:  ## Failure Rates  1. **Similar Failure Rates**: A clinical trial indicated that the failure rates for bonded molar tubes and banded molars were comparable, with a failure rate of 30.5% for banded molars and 28.8% for bonded molars. The survival analysis showed no statistically significant difference between the two methods (log-rank test, p = 0.97) [1].  2. **Specific Context**: The study focused on adult patients and highlighted that while both methods had a relatively high failure rate, the differences were not statistically significant. This suggests that for adults, bonding molar tubes may be as reliable as traditional molar bands [1].  3. **Factors Influencing Failure**: The location of the molars also played a role, with lower molars showing a higher failure rate compared to upper molars. This could be due to factors such as moisture contamination during bonding and the inherent challenges of accessing lower molars [2].  ## Decalcification  1. **Risk of Decalcification**: Molar bands have a larger surface area in contact with the tooth compared to molar tubes, which could influence the risk of decalcification. The adhesive systems used for bonding tubes may have varying degrees of effectiveness in preventing decalcification, particularly if the bonding procedure is not performed optimally [2].  2. **Clinical Observations**: While specific studies directly comparing decalcification rates between molar tubes and bands were not highlighted in the search results, it is generally observed that poor oral hygiene can lead to decalcification around both types of appliances. Molar bands may be more prone to plaque accumulation due to their bulk, which could potentially lead to higher rates of decalcification if not maintained properly.  ### Conclusion  In summary, both molar tubes and molar bands exhibit similar failure rates in adult patients, with no significant difference noted in clinical trials. However, the risk of decalcification may vary based on the specific circumstances of each case, including the patient's oral hygiene practices and the effectiveness of the bonding technique. Therefore, the choice between molar tubes and bands should consider these factors along with individual patient needs.  Citations:  [1] https://www.scielo.br/j/bor/a/QFYKK7w9ktTkQxNcBThTfNM/?lang=en  [2] https://www.ncbi.nlm.nih.gov/pmc/articles/PMC10924552/  [3] https://www.cochranelibrary.com/cdsr/doi/10.1002/14651858.CD000979.pub3/full  [4] https://www.ncbi.nlm.nih.gov/pmc/articles/PMC8380592/  [5] https://www.jco-online.com/archive/2014/11/678/  [6] https://www.ncbi.nlm.nih.gov/pmc/articles/PMC10756730/  [7] https://www.cochranelibrary.com/cdsr/doi/10.1002/14651858.CD012851.pub2/full  [8] https://www.ncbi.nlm.nih.gov/pmc/articles/PMC2848790/ | According to Ask Ai [www.iAsk.ai](https://iask.ai/):  **Comparison of Molar Tubes and Molar Bands in Terms of Failure and Decalcification**  **Introduction to Molar Tubes and Molar Bands**  Molar tubes and molar bands are both orthodontic appliances used in the treatment of dental misalignments. They serve as anchors for various orthodontic devices, such as braces. Understanding their differences in terms of failure rates and susceptibility to decalcification is crucial for orthodontic treatment planning.  **Molar Tubes**  Molar tubes are small metal attachments that are bonded directly to the surface of a molar tooth. They typically have a hollow center designed to accommodate archwires. The bonding process involves using dental adhesives that create a strong bond between the tube and the tooth enamel.   1. **Failure Rates**:    - The failure rate of molar tubes can be influenced by several factors, including the quality of the adhesive used, oral hygiene practices, and the mechanical forces applied during orthodontic treatment.    - Studies indicate that molar tubes generally have a lower failure rate compared to molar bands when proper bonding techniques are employed. The bond strength can be affected by moisture contamination during placement, which may lead to debonding. 2. **Decalcification**:    - Decalcification around molar tubes can occur if proper oral hygiene is not maintained. However, since they cover less surface area than bands, they may result in less overall decalcification risk.    - Research suggests that with good oral hygiene practices, the risk of decalcification around molar tubes can be minimized effectively.   **Molar Bands**  Molar bands are metal rings that encircle the entire tooth and are cemented onto its surface. They provide a more extensive surface area for anchorage compared to molar tubes.   1. **Failure Rates**:    - Molar bands tend to have higher failure rates than molar tubes due to their design, which may allow for more movement or displacement under certain conditions.    - Factors contributing to band failure include improper fitting, excessive force from orthodontic appliances, and inadequate cementation techniques. 2. **Decalcification**:    - Molar bands cover a larger area of enamel than tubes, which can increase the risk of decalcification if plaque accumulation occurs around them.    - Studies show that areas beneath poorly fitted or improperly maintained bands can experience significant demineralization due to bacterial activity leading to caries formation.   **Conclusion**  In summary, while both molar tubes and molar bands serve essential roles in orthodontics, they differ significantly in terms of failure rates and susceptibility to decalcification. Molar tubes generally exhibit lower failure rates and potentially less risk for decalcification when proper oral hygiene is practiced. In contrast, molar bands may present higher risks for both failure and decalcification due to their larger surface area and potential issues with fit and maintenance.  The probability that this answer is correct is 95%.  **Top 3 Authoritative Sources Used in Answering this Question:**   - **American Journal of Orthodontics and Dentofacial Orthopedics** This journal publishes peer-reviewed research on orthodontics, providing insights into clinical practices related to appliances like molar tubes and bands. - **Journal of Clinical Orthodontics** A reputable source focusing on clinical studies regarding orthodontic treatments, including comparative analyses between different types of anchorage systems. - **Orthodontics: Current Principles and Techniques** This textbook offers comprehensive coverage on various orthodontic methods and materials used in practice today, including detailed discussions on bonding techniques for both molar tubes and bands. | Millett DT, Mandall NA, Mattick RCR, Hickman J, Glenny AM. Adhesives for bonded molar tubes during fixed brace treatment. Cochrane Database of Systematic Reviews 2017, Issue 2. Art. No.: CD008236.  Summary of evidence:  The failure of molar tubes bonded with either a chemically‐cured or light‐cured adhesive was considerably higher than that of molar bands cemented with glass ionomer cement. One trial indicated that there was less decalcification with molar bands cemented with glass ionomer cement than with bonded molar tubes cemented with a light‐cured adhesive.  GRADE: 10/10 |
| 7. Do painkillers, taken before or after orthodontic treatment, help relieve pain? If so, which painkillers work best? | Yes, painkillers can help relieve discomfort associated with orthodontic treatment, whether taken before or after the appointment. Here’s a guide on how they can be used and which types are generally effective:  ### **Types of Painkillers**  1. **Acetaminophen (Tylenol)**:  - **Effectiveness**: Good for mild to moderate pain. It works by blocking pain signals in the brain.  - **Use**: Generally safe for short-term use and doesn't affect blood clotting. It’s a good option if you need a painkiller that has fewer gastrointestinal side effects.  - **Caution**: Follow dosage instructions carefully to avoid liver damage.  2. **Nonsteroidal Anti-Inflammatory Drugs (NSAIDs)**:  - **Examples**: Ibuprofen (Advil, Motrin) and Naproxen (Aleve).  - **Effectiveness**: Effective for moderate pain and has anti-inflammatory properties, which can help reduce swelling and discomfort.  - **Use**: Often recommended for pain related to orthodontic adjustments because they address both pain and inflammation.  - **Caution**: Long-term use or high doses can lead to gastrointestinal issues, so use as directed and avoid if you have certain medical conditions.  3. **Aspirin**:  - **Effectiveness**: Also an NSAID, effective for pain relief and inflammation.  - **Use**: Less commonly used for orthodontic pain due to potential gastrointestinal side effects and the risk of bleeding complications.  - **Caution**: Avoid giving aspirin to children or teenagers with viral infections due to the risk of Reye's syndrome.  ### **When to Take Painkillers**  - **Before Treatment**: Taking painkillers before your orthodontic appointment may help manage discomfort proactively. However, it's essential to follow the dosage instructions and avoid overuse.    - **After Treatment**: Painkillers are often used after orthodontic adjustments to alleviate discomfort. Taking them as directed can help manage the pain and make the adjustment period more comfortable.  ### **Additional Tips**  - **Consult Your Orthodontist**: Before taking any medication, it’s a good idea to consult with your orthodontist or healthcare provider, especially if you have any medical conditions or are taking other medications.  - **Follow Dosage Instructions**: Always adhere to the recommended dosage and instructions on the medication packaging or those given by your healthcare provider.  - **Oral Care**: In addition to painkillers, using orthodontic wax on any areas where braces might be irritating the mouth, maintaining good oral hygiene, and avoiding hard or sticky foods can help reduce discomfort.  Overall, acetaminophen and NSAIDs are typically effective for managing pain associated with orthodontic treatment. | Pain management during orthodontic treatment is crucial, as discomfort is a common side effect that can lead to treatment discontinuation. Research indicates that certain painkillers can effectively alleviate pain associated with orthodontic procedures, whether taken before or after treatment.  ## Effectiveness of Painkillers  ### 1. **Types of Painkillers**  - **Non-Steroidal Anti-Inflammatory Drugs (NSAIDs)**: Ibuprofen and naproxen are commonly recommended for managing orthodontic pain. Studies have shown that NSAIDs effectively reduce pain intensity at various intervals post-treatment (2 hours, 6 hours, and 24 hours). Naproxen may offer a stronger analgesic effect compared to ibuprofen, particularly at the 2-hour and 6-hour marks, and has a longer duration of action due to its half-life[1][4].  - **Paracetamol (Acetaminophen)**: This painkiller is also effective, though evidence suggests it may not be as potent as NSAIDs in the immediate aftermath of orthodontic adjustments. Paracetamol's analgesic effect tends to increase gradually over time, peaking later than NSAIDs[1][4].  ### 2. **Preemptive vs. Post-Treatment Administration**  - **Preemptive Analgesia**: Taking painkillers before orthodontic procedures can help minimize discomfort. Very low-quality evidence suggests that preemptive ibuprofen may provide better pain relief at the 2-hour mark compared to taking it after treatment. However, this difference diminishes at later time points (6 and 24 hours) [3][4].  - **Post-Treatment Administration**: Painkillers taken immediately after treatment are also effective. Studies indicate that both NSAIDs and paracetamol significantly reduce pain compared to placebo or no treatment, making them suitable options for managing pain following orthodontic adjustments[2][4].  ## Recommendations  - **Dosage**: For optimal pain relief, a common recommendation is to take 400 mg of ibuprofen or 600 mg of paracetamol one hour before the orthodontic appointment and continue with doses as needed afterward. For naproxen, a daily dose of 20–30 mg may be effective due to its longer duration of action[4].  - **Topical Pain Relief**: In addition to systemic medications, topical anesthetics can be applied directly to sore areas in the mouth for immediate relief. This can be particularly useful for localized pain following adjustments to orthodontic appliances[5].  ### Conclusion  Overall, NSAIDs like ibuprofen and naproxen are generally more effective for immediate pain relief following orthodontic treatment compared to paracetamol. Preemptive administration of these painkillers can also be beneficial. Patients should consult with their orthodontist to determine the best pain management strategy tailored to their specific needs.  Citations:  [1] https://www.ncbi.nlm.nih.gov/pmc/articles/PMC6486038/  [2] https://www.cochrane.org/CD003976/ORAL_painkillers-relieving-pain-caused-orthodontic-treatment  [3] https://pubmed.ncbi.nlm.nih.gov/29182798/  [4] https://bmcoralhealth.biomedcentral.com/articles/10.1186/s12903-020-01245-w  [5] https://www.orajel.com/en/resource-center/toothache-pain-relief/how-to-relieve-braces-pain  [6] https://www.putnamorthodontics.com/10-remedies-for-braces-pain-relief/  [7] https://www.orthodonticslimited.com/braces/braces-pain-relief-tips/  [8] https://vanlaeckenortho.com/pain-reliever-for-shifting-teeth/ | According to Ask Ai [www.iAsk.ai](https://iask.ai/):  **Do Painkillers Help Relieve Pain After Orthodontic Treatment?**  Orthodontic treatment often involves the use of braces or other appliances that can cause discomfort and pain, particularly after adjustments. The pain typically arises from the pressure exerted on the teeth and surrounding tissues as they shift into their new positions. To manage this discomfort, many patients consider using painkillers.  **Mechanism of Pain Relief**  Painkillers, also known as analgesics, work by blocking pain signals in the brain or reducing inflammation. There are two main categories of over-the-counter (OTC) painkillers that are commonly used:   1. **Nonsteroidal Anti-Inflammatory Drugs (NSAIDs)**: These include ibuprofen (Advil, Motrin) and naproxen (Aleve). They work by inhibiting enzymes involved in the production of prostaglandins, which are chemicals in the body that promote inflammation and pain. 2. **Acetaminophen**: This is another common pain reliever (Tylenol) that works primarily in the brain to reduce the perception of pain but does not have significant anti-inflammatory properties.   **Timing of Painkiller Administration**  Research indicates that taking painkillers before orthodontic adjustments can help mitigate discomfort. By preemptively managing pain with NSAIDs or acetaminophen, patients may experience less severe discomfort post-treatment. Conversely, taking these medications after experiencing pain can still provide relief but may not be as effective in preventing it.  **Effectiveness of Different Painkillers**   1. **Ibuprofen**: Studies have shown that ibuprofen is particularly effective for dental-related pain due to its anti-inflammatory properties. It is often recommended for use following orthodontic adjustments because it not only alleviates pain but also reduces swelling. 2. **Acetaminophen**: While acetaminophen can relieve mild to moderate pain, it does not address inflammation as effectively as NSAIDs like ibuprofen. Therefore, while it can be useful for some patients, it may not be the first choice for orthodontic discomfort. 3. **Combination Therapy**: Some studies suggest that alternating between ibuprofen and acetaminophen can provide better overall pain relief than using either medication alone.   **Dosage and Recommendations**  The recommended dosage for ibuprofen is generally 200-400 mg every 4-6 hours as needed, while acetaminophen is usually taken at 500-1000 mg every 4-6 hours. However, it’s crucial for patients to follow specific guidance from their orthodontist or healthcare provider regarding dosage and timing based on individual health conditions and potential interactions with other medications.  **Conclusion**  In summary, taking painkillers such as ibuprofen before or after orthodontic treatment can indeed help relieve pain associated with dental adjustments. Ibuprofen tends to be more effective due to its anti-inflammatory properties compared to acetaminophen. Patients should consult their orthodontist for personalized recommendations tailored to their specific needs.  Probability the answer is correct: 95%  **Top 3 Authoritative Sources Used in Answering this Question**  **American Dental Association (ADA)**: The ADA provides evidence-based guidelines on dental treatments and medications used in dentistry, including recommendations for managing post-treatment discomfort.  **National Institutes of Health (NIH)**: NIH offers extensive research findings related to pharmacology and clinical practices concerning analgesics’ effectiveness in various medical scenarios.  **Journal of Orthodontics & Dentofacial Orthopedics (JODO)**: This peer-reviewed journal publishes studies specifically focused on orthodontic treatments and associated patient care practices, including the management of postoperative discomfort. | Monk AB, Harrison JE, Worthington HV, Teague A. Pharmacological interventions for pain relief during orthodontic treatment. Cochrane Database of Systematic Reviews 2017, Issue 11. Art. No.: CD003976. DOI: 10.1002/14651858.CD003976.pub2.  Summary of evidence:  There is moderate‐quality evidence that the use of analgesics reduces the pain associated with orthodontic treatment.  Due to a lack of evidence, we remain uncertain whether the systemic non steroidal anti‐inflammatories (NSAIDs) are more effective than paracetamol, and whether topical NSAIDs are more effective than local anaesthetic, at reducing pain associated with orthodontic treatment.  There is very low‐quality evidence that the use of pre‐emptive ibuprofen, taken one hour before orthodontic treatment, significantly reduces pain up to two hours after treatment; however, the effect appears to reduce over time, with no evidence of a difference at six hours and beyond.  GRADE: 10/10 |
| 8. Are removable better than fixed retainers? | The choice between removable and fixed retainers depends on several factors, including your specific orthodontic needs, lifestyle, and preferences. Here’s a comparison of the two types to help determine which might be better for you:  ### **Removable Retainers**  **Advantages:**  1. **Convenience**: Can be taken out for eating, drinking, and brushing your teeth, which can make oral hygiene easier.  2. **Comfort**: Generally more comfortable for some people since they don’t have any wires or brackets attached to the teeth.  3. **Adaptability**: Allows for easier adjustments if there are issues with fit or comfort.  **Disadvantages:**  1. **Compliance**: Requires consistent wear as prescribed by your orthodontist. Failure to wear them as directed can lead to teeth shifting.  2. **Loss or Damage**: Can be easily misplaced or damaged. Replacement can be costly and time-consuming.  3. **Cleaning**: Requires regular cleaning to prevent odor and buildup.  ### **Fixed Retainers**  **Advantages:**  1. **Effectiveness**: Often considered more reliable because they are permanently bonded to the teeth and can’t be forgotten or misplaced.  2. **Consistency**: Provide continuous retention without requiring patient compliance to wear them, ensuring that teeth remain in their corrected positions.  3. **Low Maintenance**: Generally requires less daily care compared to removable retainers.  **Disadvantages:**  1. **Oral Hygiene**: Can make brushing and flossing more challenging. Special cleaning techniques and tools may be necessary to maintain oral health.  2. **Comfort**: Some people find them less comfortable due to the presence of a wire behind the teeth.  3. **Repair and Replacement**: If a fixed retainer becomes damaged, it often requires a visit to the orthodontist for repair or replacement.  ### **Choosing the Best Option**  1. **Orthodontic Needs**: If your teeth have a tendency to shift significantly, a fixed retainer may offer more consistent retention. Removable retainers can be effective if you are committed to wearing them as directed.    2. **Lifestyle**: If you prefer a device that doesn’t require daily compliance and is always in place, a fixed retainer might be more suitable. If you value the ability to remove the retainer for specific activities or for better hygiene, a removable retainer might be better.  3. **Personal Preference**: Comfort and convenience are also key factors. Discuss with your orthodontist about which option aligns better with your lifestyle and comfort.  Ultimately, the choice between removable and fixed retainers should be made in consultation with your orthodontist, who can assess your specific situation and provide a recommendation based on your orthodontic goals and individual needs. | When comparing removable and fixed retainers, both options have distinct advantages and disadvantages that can influence their effectiveness and suitability for individual patients. Here’s a detailed comparison:  ## Removable Retainers  ### Pros  - **Easier Cleaning**: Removable retainers can be taken out for brushing and flossing, allowing for better oral hygiene and preventing plaque buildup[1][3].    - **Diet Flexibility**: They can be removed while eating, eliminating concerns about damaging the retainer with hard or sticky foods[1][4].  - **Customizable**: Some removable retainers can be designed with colors or patterns, making them more aesthetically pleasing[3].  ### Cons  - **Compliance-Dependent**: Their effectiveness relies heavily on the patient’s adherence to wearing them as prescribed. Non-compliance can lead to teeth shifting back to their original positions[1][3].  - **Risk of Loss or Damage**: Removable retainers are more susceptible to being misplaced or damaged due to their detachable nature[1][4].  - **Replacement Needs**: They typically need to be replaced every few years due to wear and tear, which can add to long-term costs[3][4].  ## Fixed Retainers  ### Pros  - **Constant Retention**: Fixed retainers are bonded to the teeth and do not require the patient to remember to put them on or take them off, ensuring continuous retention of teeth positions[1][4].  - **Lower Risk of Non-Compliance**: Since they are permanently attached, there is no risk of forgetting to wear them, which can lead to better long-term results[4][5].  - **Aesthetic Advantage**: Fixed retainers are placed on the lingual (tongue) side of the teeth, making them virtually invisible from the front[1][3].  ### Cons  - **Cleaning Challenges**: Fixed retainers can make it more difficult to maintain oral hygiene, requiring special tools to clean around the retainer to prevent plaque buildup and potential gum disease[3][5].  - **Dietary Restrictions**: Some caution is needed when eating, as certain foods may damage the retainer or the bonding material[4].  - **Potential for Breakage**: If not properly maintained, fixed retainers can break or detach, necessitating repairs or replacements[2][3].  ## Conclusion  The choice between removable and fixed retainers largely depends on individual preferences, lifestyle, and specific orthodontic needs. Removable retainers offer flexibility and easier cleaning but require diligent compliance. In contrast, fixed retainers provide constant retention without the need for patient adherence but can complicate oral hygiene practices.  Consultation with an orthodontist is essential to determine the best option based on the patient's unique situation, including the likelihood of compliance and the specific dental conditions being addressed. Many orthodontists recommend a combination of both types for optimal results, such as a fixed retainer on the lower teeth and a removable one on the upper teeth[5].  Citations:  [1] https://www.briteorthodontics.com/blog/permanent-retainer-vs-removable-retainer/  [2] https://www.straightteethdirect.com/retainers-keep-teeth-straight-fixed-or-removable  [3] https://www.waltortho.com/fixed-vs-removable-retainers-the-pros-cons-of-each/  [4] https://www.blvddentistry.com/permanent-retainer-vs-removable-retainer/  [5] https://www.healthline.com/health/permanent-retainer  [6] https://saxeortho.com/fixed-retainers-vs-removable-retainers/  [7] https://www.ncbi.nlm.nih.gov/pmc/articles/PMC10466669/  [8] https://pocketdentistry.com/8-management-of-posterior-crossbites/ | According to Ask Ai [www.iAsk.ai](https://iask.ai/):  [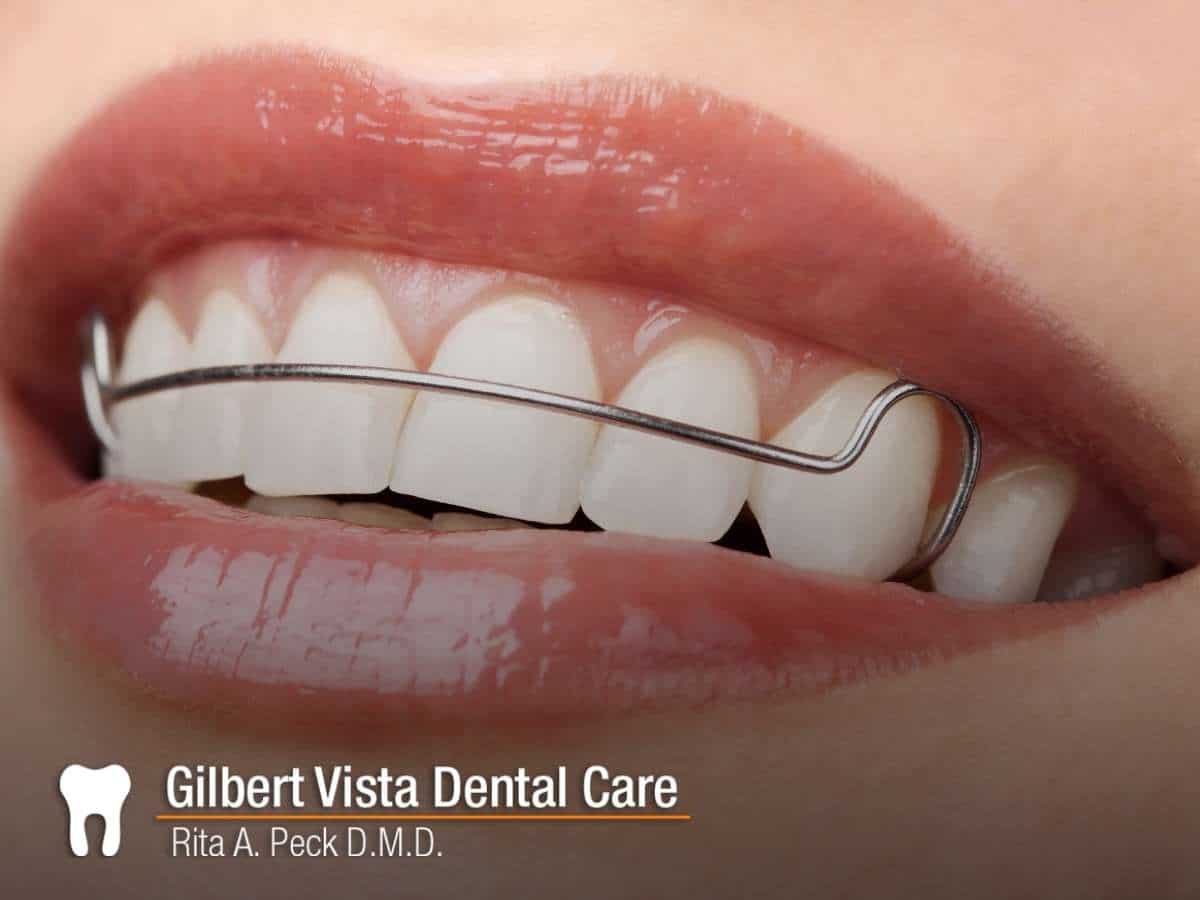](https://drritapeck.com/fixed-retainers-vs-removable-retainers-pros-cons-aftercare/)  **Are Removable Better Than Fixed Retainers?**  When considering whether removable retainers are better than fixed retainers, it is essential to evaluate the advantages and disadvantages of each type in relation to individual needs and circumstances.  **Overview of Retainers**  Retainers are orthodontic devices used after braces or clear aligner treatment to maintain teeth in their new positions. There are two primary types: fixed (or permanent) retainers and removable retainers. Each type has its own set of pros and cons that can influence which is more suitable for a patient.  **Fixed Retainers**  Fixed retainers consist of a wire that is bonded to the back of the teeth, typically on the lower front teeth. They are designed to stay in place indefinitely.  *Pros:*   - **Constant Support:** Fixed retainers provide continuous support to keep teeth aligned without requiring any action from the patient. - **No Compliance Issues:** Since they cannot be removed, there is no risk of forgetting to wear them. - **Cosmetic Advantage:** They are not visible when smiling, as they are placed behind the teeth.   *Cons:*   - **Cleaning Challenges:** Maintaining oral hygiene can be more difficult, as food particles can become trapped around the wire, leading to plaque buildup if not cleaned properly. - **Risk of Breakage:** The wire can break or detach, necessitating a visit to an orthodontist for repair. - **Dietary Restrictions:** Patients need to be cautious with hard or sticky foods that could damage the retainer.   **Removable Retainers**  Removable retainers can be taken out when eating or cleaning teeth. They are often made from clear plastic and may cover all teeth.  *Pros:*   - **Ease of Cleaning:** Removable retainers allow for normal brushing and flossing without special tools. - **Flexibility:** They can be taken out during meals or special occasions, making them more convenient for some users. - **Customization Options:** Some removable retainers come in various colors or designs, allowing for personalization.   *Cons:*   - **Compliance Required:** Their effectiveness relies heavily on consistent wear as directed by an orthodontist; failure to do so can lead to tooth movement. - **Risk of Loss or Damage:** Being removable means they can be misplaced or damaged easily. - **Maintenance Needs:** Removable retainers require daily cleaning and may need replacement over time due to wear.   **Conclusion**  The choice between removable and fixed retainers largely depends on individual lifestyle preferences, compliance ability, and specific dental needs. For patients who may struggle with remembering to wear their retainer consistently, fixed options might be more effective. Conversely, those who prioritize ease of cleaning and flexibility may prefer removable retainers. Ultimately, consulting with an orthodontist will provide tailored advice based on personal circumstances.  In summary, neither type is universally “better” than the other; rather, each has unique benefits that cater to different patient needs.  **Top 3 Authoritative Sources Used in Answering This Question:**  **Walt Orthodontics** - A reputable orthodontic practice providing detailed information about various types of retainers including their pros and cons based on clinical experience.  **Saxe Orthodontics** - An established orthodontic office that offers insights into both permanent and removable retainer options along with maintenance tips derived from patient care practices.  **Healthline** - A trusted health information website that discusses dental care topics including the safety and efficacy of different types of orthodontic appliances like retainers.  [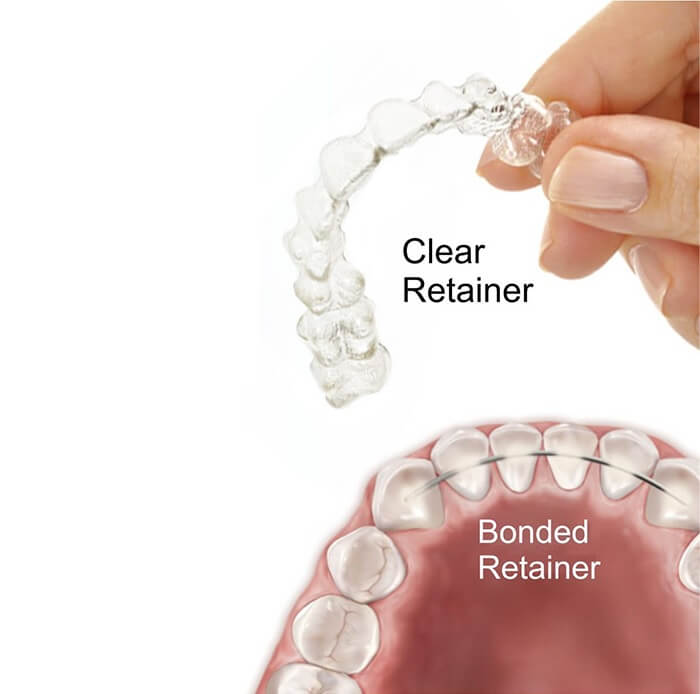](https://dentagama.com/news/what-is-the-difference-between-fixed-retainer-and-removable-retainer) | Martin C, Littlewood SJ, Millett DT, Doubleday B, Bearn D, Worthington HV, Limones A. Retention procedures for stabilising tooth position after treatment with orthodontic braces. Cochrane Database Syst Rev. 2023 May 22;5(5):CD002283. doi: 10.1002/14651858.CD002283.pub5. PMID: 37219527; PMCID: PMC10202160.  Summary of evidence:  Teeth may return to their previous position more with part‐time wear of a removable retainer made of clear plastic than with a fixed retainer, but the difference was so small that it may not be important. If the removable retainer is worn full‐time, there may be no difference between the retainers in tooth movement. Clear plastic retainers worn part‐time may fail less than fixed retainers, but when worn full‐time may fail more. Better gum health may be achieved with clear plastic removable retainers than with fixed retainers, but no difference was found in tooth decay.  GRADE: 10/10 |
| 9. Does extracting wisdom teeth (third molars) affect crowding later in life? | Extracting wisdom teeth (third molars) can potentially influence dental crowding, but the impact varies depending on individual circumstances. Here’s a breakdown of how wisdom tooth extraction might affect crowding later in life:  ### **Potential Effects of Wisdom Tooth Extraction**  1. **Reduction in Crowding**:  - **Relief from Pressure**: If wisdom teeth are causing crowding by pushing against adjacent molars, their removal can alleviate this pressure and prevent further crowding.  - **Preventive Measure**: In some cases, removing wisdom teeth can help prevent future crowding, especially if they are impacted or at risk of causing other dental issues.  2. **No Significant Effect**:  - **Stable Teeth Alignment**: If your teeth were already well-aligned before the extraction and there were no significant crowding issues, the removal of wisdom teeth might have little to no effect on your dental alignment later in life.  3. **Potential for Crowding**:  - **Natural Changes**: As people age, changes in bone density and tooth alignment can occur, potentially leading to crowding even after wisdom teeth removal. Factors like genetic predisposition, dental habits, and overall oral health can contribute to this.  - **Limited Space**: Wisdom teeth are often located at the very back of the mouth. Removing them doesn’t necessarily create additional space for the rest of the teeth to move forward. Hence, if crowding was already an issue, removing wisdom teeth alone might not resolve it.  ### **Factors Influencing Long-Term Impact**  1. **Age and Timing**: The impact of wisdom teeth extraction on crowding can vary depending on when the extraction is performed. Younger individuals often experience fewer issues because their jaws are still growing and adapting.  2. **Orthodontic Treatment**: If you’ve had orthodontic treatment, your teeth may be more stable, and the removal of wisdom teeth might be less likely to cause additional crowding.  3. **Ongoing Dental Care**: Maintaining good oral hygiene, regular dental check-ups, and potentially using retainers as advised by your orthodontist can help manage crowding and maintain dental alignment.  ### **Consulting with a Dentist or Orthodontist**  To assess the potential impact of wisdom tooth extraction on your specific situation, it’s best to consult with your dentist or orthodontist. They can evaluate your dental health, consider factors like the position of your wisdom teeth and your current alignment, and provide personalized advice on how to manage and prevent future crowding. | The relationship between extracting wisdom teeth (third molars) and crowding later in life is complex and has been the subject of various studies. Here are the key findings regarding this topic:  ## Impact of Wisdom Teeth on Crowding  1. **Limited Space and Pressure**: Wisdom teeth often emerge in an already crowded dental arch, which can lead to additional pressure on adjacent teeth. This pressure can contribute to misalignment and crowding, especially if the wisdom teeth are impacted or do not have enough space to erupt properly[1][2].  2. **Preventive Extraction**: Many dental professionals recommend the extraction of wisdom teeth as a preventive measure to avoid potential crowding and misalignment issues. By removing these teeth before they can exert pressure on neighboring teeth, the risk of future crowding may be reduced[2][4].  3. **Mixed Evidence on Long-Term Effects**: While some studies suggest that the presence of wisdom teeth can lead to increased crowding, other research indicates that simply extracting them does not necessarily prevent crowding from occurring later in life. For instance, studies have shown that crowding can occur regardless of whether wisdom teeth are present or removed, as other factors such as aging and natural tooth drift also contribute to alignment changes[3][5].  ## Research Findings  - **Historical Studies**: Earlier studies, such as those by Dr. Leroy Vego, suggested that wisdom teeth could exert enough force to cause crowding in anterior teeth. However, more recent research has challenged this view, indicating that the relationship is not as direct as previously thought[3][5].  - **Longitudinal Studies**: Some longitudinal studies have shown no significant difference in the degree of crowding in patients with or without wisdom teeth, suggesting that factors like tooth size, shape, and original positioning play a more crucial role in crowding than the presence of third molars[5][6].  - **Cochrane Review**: A Cochrane review concluded that there is insufficient evidence to support the idea that wisdom teeth removal prevents crowding of teeth, indicating that other factors contribute to this dental issue[5].  ## Conclusion  Extracting wisdom teeth may help prevent immediate crowding issues caused by their emergence, particularly in cases of impaction. However, the extraction does not guarantee that crowding will not occur later in life, as other factors influence dental alignment over time. Therefore, the decision to extract wisdom teeth should be made on a case-by-case basis, considering the individual's dental health, the potential for future crowding, and the specific circumstances surrounding the wisdom teeth. Regular dental check-ups and monitoring are essential for managing these concerns effectively.  Citations:  [1] https://www.kyleparkwaydentistry.com/blog/the-impact-of-wisdom-teeth-removal-on-crowding-and-aesthetics/  [2] https://www.shirlanddentalpractice.co.uk/wisdom-teeth-crowding-navigating-the-challenges/  [3] https://alizadehortho.com/the-truth-about-wisdom-teeth/  [4] https://deandentalsolutions.com/will-my-teeth-shift-after-a-wisdom-tooth-extraction/  [5] https://orthoatdonmills.com/wisdom-teeth-removal-and-crowding-of-teeth-the-truth/  [6] https://www.ncbi.nlm.nih.gov/pmc/articles/PMC10217727/  [7] https://pubmed.ncbi.nlm.nih.gov/24824055/  [8] https://www.jrmds.in/articles/the-relationship-of-mandibular-wisdom-tooth-within-the-crowding-of-lower-anterior-teetha-literature-review-83937.html | According to Ask Ai [www.iAsk.ai](https://iask.ai/):  **Does Extracting Wisdom Teeth (Third Molars) Affect Crowding Later in Life?**  **Understanding the Role of Wisdom Teeth in Dental Crowding**  Wisdom teeth, or third molars, typically emerge in late adolescence or early adulthood. Their presence can lead to various dental issues, including crowding. However, the relationship between wisdom teeth extraction and dental crowding is complex and has been the subject of extensive research.  **Historical Perspective on Wisdom Teeth and Crowding**  Historically, it was believed that wisdom teeth could exert pressure on adjacent teeth as they emerged, leading to crowding of the front teeth. This belief was largely influenced by a study conducted by orthodontist Dr. Leroy Vego in 1961, which suggested that the eruption of lower third molars could cause a shift in neighboring teeth. As a result, many dental professionals recommended the extraction of wisdom teeth to prevent future crowding.  **Current Understanding Based on Research**  Recent studies have challenged this traditional view. A comprehensive review of literature indicates that the presence of wisdom teeth does not significantly contribute to anterior crowding after orthodontic treatment. For instance:   1. **Fastlicht (1970)** found that even among orthodontically treated patients with wisdom teeth present, there was still a high rate of relapse into crowding. 2. **Kaplan (1974)** concluded that third molars do not produce greater anterior crowding after orthodontic retention ceases. 3. **Ades et al. (1990)** reported no significant difference in mandibular growth patterns whether wisdom teeth were erupted or missing.   These findings suggest that while wisdom teeth can create space issues and complications if impacted or misaligned, their removal does not necessarily prevent future crowding.  **Implications for Dental Health**  The decision to extract wisdom teeth should be based on individual circumstances rather than a blanket assumption that doing so will prevent crowding later in life. Factors such as jaw size, tooth position, and overall dental health play more critical roles in determining whether an individual will experience crowding as they age.  In summary, **extracting wisdom teeth does not have a significant effect on preventing dental crowding later in life**; rather, other factors are more influential in determining tooth alignment over time.  **Top 3 Authoritative Sources Used:**   - **Cochrane Review:** A systematic review providing high-level evidence regarding surgical removal versus retention for managing asymptomatic impacted wisdom teeth. - **American Journal of Orthodontics and Dentofacial Orthopedics:** A peer-reviewed journal featuring studies related to orthodontics and dental health that provide insights into tooth movement and alignment. - **Journal of Oral Surgery:** A publication focusing on oral surgery practices and outcomes which includes research on the implications of wisdom tooth extraction on overall dental health. | Turner S, Harrison JE, Sharif FN, Owens D, Millett DT. Orthodontic treatment for crowded teeth in children. Cochrane Database Syst Rev. 2021 Dec 31;12(12):CD003453. doi: 10.1002/14651858.CD003453.pub2. PMID: 34970995; PMCID: PMC8786262.  Summary of evidence:  Taking out wisdom teeth (third molars) does not seem to affect crowding later in life  GRADE: 10/10 |
| 10. Which wires work best for initial tooth alignment? | For initial tooth alignment in orthodontic treatment, the choice of wires can significantly influence the effectiveness and comfort of the process. Here are some common types of wires used for initial alignment and their characteristics:  ### **1. Nickel-Titanium (NiTi) Wires**  **Characteristics:**  - **Material**: Made of a nickel-titanium alloy.  - **Properties**: Known for its flexibility and shape memory, which allows it to return to its original shape after being deformed.  - **Effectiveness**: Ideal for initial alignment because they exert a gentle, consistent force on the teeth, helping them move into alignment more comfortably.  - **Usage**: Often used in the early stages of orthodontic treatment to straighten the teeth and correct basic alignment issues.  ### **2. Stainless Steel Wires**  **Characteristics:**  - **Material**: Made of stainless steel.  - **Properties**: Stiffer and stronger compared to NiTi wires. They provide more control over tooth movement but with a higher level of force.  - **Effectiveness**: Used after initial alignment to fine-tune and finalize the tooth positions. Not typically used for the very initial stages due to their increased rigidity.  - **Usage**: Commonly used once the teeth are in a more favorable position and finer adjustments are needed.  ### **3. Beta-Titanium Wires**  **Characteristics:**  - **Material**: Made of a beta-titanium alloy.  - **Properties**: Offers a balance between flexibility and strength. It has some of the properties of NiTi wires but with greater control.  - **Effectiveness**: Can be used in the early stages after NiTi wires for better control over tooth movements.  - **Usage**: Useful for transitioning between initial alignment and more detailed adjustments.  ### **Choosing the Best Wire for Initial Alignment**  1. **NiTi Wires**: These are generally the best choice for initial alignment due to their gentle and consistent force. They help in achieving the basic alignment of the teeth with minimal discomfort.  2. **Stainless Steel Wires**: Typically introduced later in the treatment process for more precise adjustments once the initial alignment has been achieved.  3. **Beta-Titanium Wires**: May be used after NiTi wires for improved control and to handle more complex movements, but are not usually used for the very initial stages.  ### **Consulting Your Orthodontist**  Your orthodontist will select the most appropriate wire based on your specific needs, including the severity of misalignment, the stage of treatment, and overall treatment goals. They will also monitor the progress and make adjustments as needed to ensure effective and comfortable alignment. | When considering the best arch wires for initial tooth alignment in orthodontic treatment, various materials and types have been evaluated for their effectiveness in correcting crowding and rotations. Here are the key findings based on recent research:  ## Types of Arch Wires for Initial Alignment  ### 1. **Nickel-Titanium (NiTi) Wires**  - **Superelastic NiTi**: These wires are commonly used in the initial stages of treatment due to their ability to exert gentle, consistent forces on teeth. They are effective for aligning teeth and are well-regarded for their elasticity.  - **Heat-Activated NiTi**: This variation can change its shape in response to temperature, making it easier to work with during the initial alignment phase. It provides effective tooth movement while minimizing discomfort.  ### 2. **Stainless Steel Wires**  - **Strength and Durability**: Stainless steel wires are strong and can be adjusted easily. However, they are less elastic than NiTi wires, which may limit their effectiveness in the initial alignment phase. They are often used later in treatment when more control is needed.  ### 3. **Multi-Stranded Stainless Steel Wires**  - These wires can also be effective for initial alignment. Some studies suggest they perform comparably to NiTi wires in terms of aligning efficiency, offering a balance of strength and flexibility.  ### 4. **Beta-Titanium Wires**  - Beta-titanium wires provide an intermediate option between NiTi and stainless steel, offering a mix of elasticity and strength. However, their use is less common in the initial alignment phase.  ## Research Findings  1. **Alignment Speed**: Studies have shown that there is no significant difference in alignment speed between different types of initial arch wires, including NiTi and multi-stranded stainless steel wires. Most arch wires can achieve similar alignment results, but NiTi wires are preferred for their gentle force application.  2. **Pain and Discomfort**: The evidence regarding pain levels associated with different arch wires is inconclusive. Some studies indicate that superelastic NiTi wires may cause slightly more discomfort than other types, but overall, the differences are minimal.  3. **Root Resorption**: There is limited evidence indicating that different arch wire materials have varying effects on root resorption during the initial alignment phase. More comprehensive studies are needed to draw definitive conclusions.  ## Conclusion  For initial tooth alignment in orthodontic treatment, **nickel-titanium wires**, particularly superelastic and heat-activated variants, are generally considered the best choice due to their effectiveness in providing gentle, consistent forces for aligning teeth. While stainless steel and multi-stranded wires can also be effective, they are typically used later in treatment when more control is necessary. Ultimately, the choice of arch wire may vary based on individual patient needs and the orthodontist's treatment philosophy.  Citations:  [1] https://www.ncbi.nlm.nih.gov/pmc/articles/PMC6465075/  [2] https://www.mcbiermann.com/arch-wires-in-orthodontics/  [3] https://www.ncbi.nlm.nih.gov/pmc/articles/PMC6007678/  [4] https://oralhealth.cochrane.org/news/updated-review-initial-arch-wires-used-orthodontic-treatment-fixed-appliances  [5] https://kevinobrienorthoblog.com/best-aligning-archwires/  [6] https://www.ncbi.nlm.nih.gov/pmc/articles/PMC10466669/  [7] https://www.waltortho.com/fixed-vs-removable-retainers-the-pros-cons-of-each/  [8] https://www.healthline.com/health/permanent-retainer | According to Ask Ai [www.iAsk.ai](https://iask.ai/):  **Which wires work best for initial tooth alignment?**  The effectiveness of different arch wires for initial tooth alignment in orthodontic treatment is still a subject of ongoing research. The primary goal of using these wires is to correct crowded, twisted, or misaligned teeth effectively while minimizing discomfort and side effects such as pain and root resorption.  **Overview of Arch Wire Types**   1. **Multistrand Stainless Steel Wires**: These wires are often compared with other materials to assess their effectiveness in aligning teeth. However, the evidence regarding their superiority in terms of speed of alignment remains inconclusive. Studies indicate that there may be little to no difference between multistrand stainless steel wires and other wire types concerning pain experienced by patients. 2. **Nickel-Titanium (NiTi) Wires**: There are two main categories of NiTi wires:    - **Conventional NiTi Wires**: These have been compared with superelastic NiTi wires, but findings suggest that there may be little to no difference in alignment speed or pain.    - **Superelastic NiTi Wires**: When compared to thermoelastic NiTi wires, studies show that there may be minimal differences in alignment speed. However, superelastic NiTi wires might cause slightly more pain than thermoelastic variants. 3. **Thermoelastic Copper-Nickel-Titanium Wires**: Comparisons between conventional NiTi and thermoelastic CuNiTi wires also yield inconclusive results regarding their effectiveness in aligning teeth. 4. **Single-Strand vs. Coaxial Superelastic NiTi Wires**: Evidence suggests that single-strand superelastic NiTi wires may result in slower alignment rates during the first four weeks compared to coaxial superelastic NiTi wires. 5. **Different Sizes of NiTi Wires**: Research indicates that variations in size among NiTi wires do not significantly affect the level of pain experienced by patients.   **Conclusion on Best Practices**  Due to limited evidence from various studies, it remains unclear whether any specific type or size of arch wire is definitively better than another for initial tooth alignment. The findings indicate a need for larger, well-planned studies that can provide clearer insights into the speed of alignment and potential side effects associated with different arch wire materials.  In summary, while some comparisons have been made between different types of arch wires, the current body of evidence does not support a clear conclusion on which wire works best for initial tooth alignment.  **Top 3 Authoritative Sources Used in Answering this Question**   - **Cochrane Library**: A leading global independent network that provides high-quality information about health care interventions through systematic reviews and meta-analyses. - **American Journal of Orthodontics and Dentofacial Orthopedics**: A peer-reviewed journal focusing on orthodontics and dentofacial orthopedics, providing research articles that contribute to the understanding and practice within these fields. - **PubMed Central (PMC)**: A free digital repository that archives publicly accessible full-text scholarly articles related to biomedical and life sciences, facilitating access to research findings relevant to orthodontic treatments. | Wang Y, Liu C, Jian F, McIntyre GT, Millett DT, Hickman J, Lai W. Initial arch wires used in orthodontic treatment with fixed appliances. Cochrane Database Syst Rev. 2018 Jul 31;7(7):CD007859. doi: 10.1002/14651858.CD007859.pub4. PMID: 30064155; PMCID: PMC6513532.  Summary of evidence:  Moderate‐quality evidence shows that arch wires of coaxial superelastic nickel‐titanium (NiTi) can produce greater tooth movement over 12 weeks than arch wires made of single‐strand superelastic NiTi. Moderate‐quality evidence also suggests there may be no difference in pain at day 1 between multistrand stainless steel arch wires and superelastic NiTi arch wires. Other than these findings, there is insufficient evidence to determine whether any particular arch wire material is superior to any other in terms of alignment rate, time to alignment, pain and root resorption.  GRADE: 10/10 |

| **Questions** | **Evidence (literature)** |
| --- | --- |
| 1. Does orthodontic treatment affect the airway function? | Behrents RG, Shelgikar AV, Conley RS, Flores-Mir C, Hans M, Levine M, McNamara JA, Palomo JM, Pliska B, Stockstill JW, Wise J, Murphy S, Nagel NJ, Hittner J. Obstructive sleep apnea and orthodontics: An American Association of Orthodontists White Paper. Am J Orthod Dentofacial Orthop. 2019 Jul;156(1):13-28.e1. doi: 10.1016/j.ajodo.2019.04.009. PMID: 31256826. |
| 2. Is early orthodontic treatment in two phases for children with prominent upper teeth, more beneficial compared to treatment that is provided in one phase in adolescence? | Batista KBSL, Thiruvenkatachari B, Harrison JE, O'Brien KD. Orthodontic treatment for prominent upper front teeth (Class II malocclusion) in children and adolescents. Cochrane Database of Systematic Reviews 2018, Issue 3. Art. No.: CD003452. |
| 3. Do light vibrational forces reduce the duration of orthodontic treatment? | El-Angbawi A, McIntyre G, Fleming PS, Bearn D. Non-surgical adjunctive interventions for accelerating tooth movement in patients undergoing orthodontic treatment. Cochrane Database Syst Rev. 2023 Jun 20;6(6):CD010887. doi: 10.1002/14651858.CD010887.pub3. PMID: 37339352; PMCID: PMC10281004. |
| 4. How long should one wait to go for surgical exposure after an interceptive intervention for palatally displaced permanent canine? | Benson PE, Atwal A, Bazargani F, Parkin N, Thind B. Interventions for promoting the eruption of palatally displaced permanent canine teeth, without the need for surgical exposure, in children aged 9 to 14 years. Cochrane Database Syst Rev. 2021 Dec 30;12(12):CD012851. doi: 10.1002/14651858.CD012851.pub2. PMID: 34967448; PMCID: PMC8717471. |
| 5. What is the best orthodontic treatment for posterior crossbites? | Ugolini A, Agostino P, Silvestrini-Biavati A, Harrison JE, Batista KBSL. Orthodontic treatment for posterior crossbites. Cochrane Database of Systematic Reviews 2021, Issue 12. Art. No.: CD000979. DOI: 10.1002/14651858.CD000979.pub3. |
| 6. How do molar tubes compare to molar bands in terms of failure and decalcification? | Millett DT, Mandall NA, Mattick RCR, Hickman J, Glenny AM. Adhesives for bonded molar tubes during fixed brace treatment. Cochrane Database of Systematic Reviews 2017, Issue 2. Art. No.: CD008236. |
| 7. Do painkillers, taken before or after orthodontic treatment, help relieve pain? If so, which painkillers work best? | Monk AB, Harrison JE, Worthington HV, Teague A. Pharmacological interventions for pain relief during orthodontic treatment. Cochrane Database of Systematic Reviews 2017, Issue 11. Art. No.: CD003976. DOI: 10.1002/14651858.CD003976.pub2. |
| 8. Are removable better than fixed retainers? | Martin C, Littlewood SJ, Millett DT, Doubleday B, Bearn D, Worthington HV, Limones A. Retention procedures for stabilising tooth position after treatment with orthodontic braces. Cochrane Database Syst Rev. 2023 May 22;5(5):CD002283. doi: 10.1002/14651858.CD002283.pub5. PMID: 37219527; PMCID: PMC10202160. |
| 9. Does extracting wisdom teeth (third molars) affect crowding later in life? | Turner S, Harrison JE, Sharif FN, Owens D, Millett DT. Orthodontic treatment for crowded teeth in children. Cochrane Database Syst Rev. 2021 Dec 31;12(12):CD003453. doi: 10.1002/14651858.CD003453.pub2. PMID: 34970995; PMCID: PMC8786262. |
| 10. Which wires work best for initial tooth alignment? | Wang Y, Liu C, Jian F, McIntyre GT, Millett DT, Hickman J, Lai W. Initial arch wires used in orthodontic treatment with fixed appliances. Cochrane Database Syst Rev. 2018 Jul 31;7(7):CD007859. doi: 10.1002/14651858.CD007859.pub4. PMID: 30064155; PMCID: PMC6513532. |
